# Investigating the Role of Spermidine in a Model System of Alzheimer’s Disease Using Correlative Microscopy and Super-resolution Techniques

**DOI:** 10.3389/fcell.2022.819571

**Published:** 2022-05-17

**Authors:** D. Lumkwana, C. Peddie, J. Kriel, L. L. Michie, N. Heathcote, L. Collinson, C. Kinnear, B. Loos

**Affiliations:** ^1^ Microscopy and Imaging Translational Technology Platform, Cancer Research UK, University College London, London, United Kingdom; ^2^ Science Technology Platform, Electron Microscopy, Francis Crick Institute, London, United Kingdom; ^3^ Central Analytical Facilities, Electron Microscopy Unit, Stellenbosch University, Stellenbosch, South Africa; ^4^ Department of Physiological Sciences, Stellenbosch University, Stellenbosch, South Africa; ^5^ DST/NRF Centre of Excellence in Biomedical Tuberculosis Research, SAMRC Centre for Tuberculosis Research, Division of Molecular Biology and Human Genetics, Faculty of Medicine and Health Sciences, Stellenbosch University, Cape Town, South Africa

**Keywords:** spermidine, autophagy, Alzheimer’s disease, correlative light and electron microscopy, super-resolution structured illumination, direct stochastic optical reconstruction microscopy, focused ion beam scanning electron microscopy

## Abstract

**Background:** Spermidine has recently received major attention for its potential therapeutic benefits in the context of neurodegeneration, cancer, and aging. However, it is unclear whether concentration dependencies of spermidine exist, to differentially enhance autophagic flux. Moreover, the relationship between low or high autophagy activity relative to basal neuronal autophagy flux and subsequent protein clearance as well as cellular toxicity has remained largely unclear.

**Methods:** Here, we used high-resolution imaging and biochemical techniques to investigate the effects of a low and of a high concentration of spermidine on autophagic flux, neuronal toxicity, and protein clearance in *in vitro* models of paraquat (PQ) induced neuronal toxicity and amyloid precursor protein (APP) overexpression, as well as in an *in vivo* model of PQ-induced rodent brain injury.

**Results:** Our results reveal that spermidine induces autophagic flux in a concentration-dependent manner, however the detectable change in the autophagy response critically depends on the specificity and sensitivity of the method employed. By using correlative imaging techniques through Super-Resolution Structured Illumination Microscopy (SR-SIM) and Focused Ion Beam Scanning Electron Microscopy (FIB-SEM), we demonstrate that spermidine at a low concentration induces autophagosome formation capable of large volume clearance. In addition, we provide evidence of distinct, context-dependent protective roles of spermidine in models of Alzheimer’s disease. In an *in vitro* environment, a low concentration of spermidine protected against PQ-induced toxicity, while both low and high concentrations provided protection against cytotoxicity induced by APP overexpression. In the *in vivo* scenario, we demonstrate brain region-specific susceptibility to PQ-induced neuronal toxicity, with the hippocampus being highly susceptible compared to the cortex. Regardless of this, spermidine administered at both low and high dosages protected against paraquat-induced toxicity.

**Conclusions:** Taken together, our results demonstrate that firstly, administration of spermidine may present a favourable therapeutic strategy for the treatment of Alzheimer’s disease and secondly, that concentration and dosage-dependent precision autophagy flux screening may be more critical for optimal autophagy and cell death control than previously thought.

## 1 Introduction

Alzheimer’s disease (AD), the leading cause of dementia in the elderly (Andrieu et al., 2015), is characterised by a progressive loss of synapses and neurons in specific brain regions, such as the hippocampus and the cerebral cortex, leading to impaired cognitive function (Zare-Shahabadi et al., 2015). AD neuropathology is underpinned by two molecular hallmarks; intracellular neurofibrillary tangles (NFTs) composed of hyper-phosphorylated Tau and extracellular amyloid beta (Aβ) plaques, composed of Aβ peptides derived from the amyloid precursor protein (APP) (Nixon and Yang, 2011; Cai et al., 2012). Accumulation of toxic Aβ peptide due to increased APP cleavage or decreased Aβ degradation has been shown to be an early event in AD progression (Selkoe and Hardy, 2016). Thus, in addition to current therapeutic approaches which include the targeting of acetylcholine breakdown and glutamate production (Folch et al., 2016), enhancing the clearance of aggregate prone proteins such as Aβ and tau has received increasing attention (Galluzzi et al., 2017; Panda et al., 2019). One of the key pathways engaged in the clearance of long-lived and aggregate-prone proteins as well as damaged cytoplasmic organelles within eukaryotic cells is autophagy (Cui et al., 2016). Cargo to be degraded is initially recruited to a phagophore, which elongates to form an autophagosome, that matures, closes, and subsequently fuses with a lysosome to form an autolysosome where hydrolytic degradation takes place (Levine and Kroemer, 2008; Cai et al., 2012; Amaravadi et al., 2019). Importantly, autophagy dysfunction is highly implicated in the onset of neurodegenerative diseases (Hara et al., 2006; Komatsu et al., 2006; Lumkwana et al., 2017). The rate of protein degradation through the entire autophagy pathway, i.e., autophagic flux (Loos et al., 2014; Klionsky et al., 2016), and its precision control has received major attention as its decline is associated with cellular toxicity, degeneration, and aging (du Toit et al., 2018a). As a result, a major focus has been spent on identifying safe and effective pharmacological agents that can induce autophagy to effectively target key aspects of the molecular pathology in AD and other neurodegenerative diseases (Rubinsztein et al., 2012; Rubinsztein et al., 2015; Galluzzi et al., 2017; Menzies et al., 2017; Panda et al., 2019).

Spermidine is a naturally occurring polyamine that is produced from putrescine or a breakdown from spermine (Madeo et al., 2018). It is present in mammalian cells where its intracellular levels decline with aging (Eisenberg et al., 2009; Pucciarelli et al., 2012; Gupta et al., 2013). Studies revealed that dietary supplementation of spermidine in mice and humans increases blood polyamine concentrations (Soda et al., 2009; Soda et al., 2013). Importantly, several studies *in vitro* and *in vivo* have demonstrated that spermidine extends lifespan in an autophagy-dependent manner (Morselli et al., 2011; Minois et al., 2012; Minois et al., 2014; Pietrocola et al., 2015; Eisenberg et al., 2016; García-Prat et al., 2016) reducing aggregate prone proteins associated with neurodegeneration (Büttner et al., 2014; Wang et al., 2012). Moreover, the ability of spermidine to reduce oxidative stress and inflammation, to improve memory (Wirth et al., 2018; Wirth et al., 2019), mitochondrial function and health (Eisenberg et al., 2016; Qi et al., 2016; Fan et al., 2017), while preventing age-induced memory impairment (Gupta et al., 2013), highlights its potential use in the treatment of neurodegenerative diseases.

Although spermidine enhances autophagy and clears aggregate-prone proteins associated with neurodegeneration, the exact relationship between its concentration and the resulting effect on autophagy activity as well as protein clearance remains unclear. Moreover, whether a concentration-dependent effect on autophagy activity exists, that would result in a defined, yet distinguishable heightened autophagy flux that impacts neuronal toxicity favourably remains poorly understood. This contributes to the challenge of modulating autophagy with high precision and translational benefit (Loos et al., 2020). Hence, in this study, we examined the effect of a low and of a high concentration of spermidine on autophagic flux, by carefully dissecting autophagosome and autolysosome pool size, autophagosome flux, transition time, autophagosome volume and size. In addition, we assessed the impact of a low and of a high concentration of spermidine on AD-related neuropathology, autophagic activity, APP cluster clearance and microtubule acetylation, employing single molecule and correlative microscopy techniques in an *in vitro* and *in vivo* model of paraquat-induced neurotoxicity and a model of APP overexpression.

## 2 Materials and Methods

### 2.1 *In Vitro* Model

Murine hypothalamus-derived GT1-7 neuronal cells and stably transfected mouse neuroblastoma cell lines (N2a) expressing Swedish mutant form (Swe) of the APP695 were used in this study. GT1-7 neuronal cells were received as a gift from Professor Pamela Mellon (University of California, San Diego, United States) (Mellon et al., 1990). Cells were cultured in Dulbecco’s Modified Eagle Medium (DMEM) (#41,965-062, Gibco^®^, Life Technologies, Johannesburg, South Africa) supplemented with 10% fetal bovine serum (FBS) (#S-0615, Biochrom, Berlin, Germany), and 1% penicillin/streptomycin (PenStrep, #15240-062, Life Technologies); 100 μg/ml streptomycin and 100 U/mL penicillin, and maintained in a humidified atmosphere in the presence of 5% CO2 at 37°C. N2aSwe cells were obtained from Professor Sangram Sisodia (Sisodia et al., 1990; Lo et al., 1994) (Department of Neurobiology, University of Chicago, United States). Cells were cultured in a 1:1 mixture of DMEM and Opti-MEM Reduced Serum Media (#31, 985,047, Gibco^®^, ThermoFisher Scientific) supplemented with 5% FBS and 1% PenStrep, and maintained at 5% CO2 at 37°C. Both GT1-7 cells and N2aSwe cells were sub-cultured using trypsin (#25,200,072, Life Technologies) to dissociate adherent cells from flasks (#707,003, WhiteSci). After trypsinisation, cells were collected in 15 ml falcon tubes (#50,015, Biocom Biotech, Centurion, South Africa) and supplemented with growth media added in a 2:1 ratio. Cells were centrifuged (5804R Centrifuge, Eppendorf, Johannesburg, South Africa) at 1,500 rpm for 3 min at room temperature (RT). The supernatant was discarded, and cells were re-suspended in growth media and seeded in either T25 (#500030, Porvair, Brackenfell, South Africa), T75 (#500029, Porvair) or T175 (#500028, Porvair) culturing flasks, or 6 well (#30,006, Bio-Smart Scientific, Edgemead, Cape Town), 48-well dishes (#30,048, Bio-Smart Scientific, Edgemead, Cape Town) or 35 mm glass-bottom dishes (#P35G-1.5-14-CGRD, MatTek Corporation, United States ) for experimental purposes.

#### 2.1.1 Treatment Conditions

To establish a suitable concentration of spermidine (Spd) (#S0266, Sigma-Aldrich, MO, United States ), GT1-7 cells were treated with three different concentrations (0.1, 1 and 10 μM) for 8 h. For autophagic flux assessment, cells were treated with the vacuolar H^+^ ATPase inhibitor bafilomycin A1 (BafA1) (#B0026, LKT labs), that prevents autophagosome-lysosome fusion (Yoshimori et al., 1991). Here, cells were treated with 400 nM BafA1 (du Toit et al., 2018b) for 4 h following treatment with spermidine. To assess the protective effects of a low and of a high concentration of spermidine against paraquat-induced neuronal toxicity, GT1-7 cells were treated with 1 and 10 μM Spd for 8 h followed by exposure to 3 mM of paraquat (PQ) for 6 h. To induce APP overexpression and assess the protective effects of a low and of a high concentration of spermidine, N2aSwe cells were treated with 5 mM butyric acid (BA) (#B103500, Sigma Aldrich, MO, United States) for 24 and 48 h. 8 h prior to the end of the treatment period, cells were incubated for further 8 h with a mixture of either 5 mM BA and 1 μM Spd or 5 mM BA and 10 μM Spd. After the completion of the treatment interventions, cells were harvested for western blot analysis or prepared for microscopy analysis or cellular viability assays.

#### 2.1.2 Cellular Viability Assay

The effect of spermidine concentrations, PQ-induced cellular toxicity and APP-induced cellular toxicity on cellular viability was measured using a WST-1 assay. Briefly, GT1-7 cells and N2aSwe cells were seeded onto 48-well plates and incubated overnight in 200 ml of complete growth media. After overnight incubation, media was aspirated, and cells were treated as desired. Following treatment, WST-1 reagent was added to cell culture media at 10 μL/200 μl and incubated for 2 h at 37°C protected from light. Then, culture plates were placed in a shaking incubator (37°C, 200 RPM) and gently shaken for 2 min to dissolve the formazan crystals. Subsequently, colorimetric readings were measured at 595 nm using an EL800 universal microplate reader (BioTek Instruments Inc., VT, United States ).

#### 2.1.3 Protein Extraction and Western Blot Analysis

After treatments were completed, growth media was aspirated, and cells were rinsed three times with cold 1x PBS. Protein lysates were harvested with 200 µl RIPA lysis buffer (50 mM Tris-HCl, 1% NP-40, 0.25% Na-deoxycholate, 150 mM NaCl, 1 mM EDTA) containing 1:100 dilution of protease inhibitor cocktail (#11,873,580,001, Sigma Aldrich) and phosphatase inhibitors (1 mM PMSF, 1 mM NaF, 1 mM Na3VO4, 1 μg/ml leupeptin, 1 μg/ml aprotonin, 1 μg/ml benzamidine, and 10 μg/ml pepstatin). Cells were detached using a scraping method and cell lysates were collected, sonicated on ice using the Misonix sonicator (Fisher Scientific, Loughborough, United Kingdom, S-4000). Subsequently, cells were centrifuged at 8000 RPM (Labnet International, Edison, NJ, United States, Spectrafuge 16 M) for 10 min at 4°C. Protein content of the lysates was determined using a Direct Detect R infrared spectrometer (DDHW00010-WW, Merck). 50 mg/ml protein was mixed in 2:1 ratio in Laemli’s sample buffer (6.5 mM Tris-HCl, 2% sodium dodecyl sulphate, 5% mercaptoethanol, 10% glycerol, 0.01% Bromophenol blue, pH 6.8). Samples were boiled at 95°C for 5 min, the protein was separated on 4%–20% polyacrylamide precast gels (#5671094, CriterionTM TGX TM Midi protein gel, Biorad). Proteins were separated at 100 V for approximately 2 h in Tris/Glycine/SDS running buffer (Bio- Rad, CA, United States). Proteins were subsequently transferred onto PVDF membranes (#170-84156, Bio-Rad) with the Midi Trans-Blot^®^ Turbo Transfer kit (Bio- Rad, CA, United States) and the Trans-Blot^®^ Turbo Transfer System (#170-4155, Bio-Rad Bio-rad), using the following conditions: 120 V and 400 A for 7 min. All membranes were blocked for 1 h in 5% fat-free milk prepared in TBS-T (137 mM NaCl, 20 mM Tris, 0.1% Tween-20, pH 7.6) followed by incubation in primary antibodies [anti-LC3B (#2775, Cell Signalling, 1:1000), anti-acetylated-α-tubulin 6-11B (#23950, Santa Cruz, 1:5000), anti-APP (#2452, Cell Signalling, 1:5000), Anti-LAMP2A (#ab18528, Abcam, 1:5000)] for overnight at 4°C. Subsequently, membranes were washed with TBS-T three times for 5 min and incubated in Horse Radish Peroxidase linked antibodies [anti-Rabbit IgG (#CST7074S, Cell Signalling, 1:5000) and anti-Mouse IgG (#CST7076S, Cell Signalling, 1:5000)] for 1 h at RT. Chemiluminescent detection was carried out with Clarity ECL Substrate (Bio-Rad, CA, United States ) on the ChemiDoc MP imaging system (Bio-rad, CA, United States). The Stain-Free™ properties of the gels were used to determine the total protein intensities of each membrane, which was used for normalization of protein-specific results (Davis et al., 2018). Bio-Rad Image Lab software was used to measure the intensity of the bands.

#### 2.1.4 Plasmids and Transfections

GT1-7 cells were transfected at a confluency of 80%–85% using a Neon^®^ 10 μl Transfection Kit (#MPK1025, Thermo Fischer Scientific) according to the manufacturer’s instructions. Briefly, following trypsinization, 1,000,000 cells for a T25 vessel or 200,000 cells for 6 well plates were aliquoted out, washed in 1x PBS and spun down. The supernatant was discarded, and the pellet of cells was resuspended in a mixture of DNA plasmid [GFP-LC3-RFP-LC3ΔG or mRFP-GFP-LC3 (AddGene)] and Neon resuspension buffer in a ratio of 1:5, a total concentration of 10 μg for a T25 ml flask or 2.0 μg for a 6 well plate. Subsequently, the suspension was pipetted into a gold-plated Neon^®^ Tip using a Neon^®^ Pipette, with the tip inserted into Neon^®^ Electrolytic buffer inside the Neon^®^ Pipette Station. Following this process, cells were electroporated at 1350 V for 1 pulse lasting a duration of 30 ms and plated into T25 flasks or 6 well dishes containing fresh media (1:1 DMEM/OptiMEM with 10% FBS) and incubated for 48 h to allow transfection to take place.

#### 2.1.5 Immunocytochemistry

GT1-7 or N2aSwe cells were seeded at a density of 150,000 cells on sterile coverslips in 6 well plates or 35 mm glass-bottom culture dishes and incubated overnight. Following incubation, cells were treated and fixed with 1:1 ratio of 4% v/v formaldehyde (FA) and growth media for 30 min. Subsequently, cells were rinsed 3 times with 1x PBS-A (in 1 L: 8 g NaCl, 0.20 g KCl, 2.20 g Na2HPO4.7H2O, 0.20 g KH2PO4 dissolved in dH2O, pH 7.4) and permeabilised with 0.2% Triton X-100 for 2 min. Cells were rinsed three times with 1x PBS-A, blocked with 5% v/v donkey serum for 30 min, at RT, before antibody staining. Cells were incubated overnight at 4°C in either anti-alpha (α)/beta (β)-tubulin rabbit, or anti-acetylated-α tubulin mouse, or anti- APP rabbit, diluted in 3% BSA (1:200). Thereafter, cells were rinsed three times with 1x PBS-A and then incubated for 50 min at RT in secondary antibodies; Alexa Fluor 568 donkey anti-rabbit, Alexa Fluor 488 donkey anti-mouse and Alexa Fluor 568 donkey anti-rabbit, respectively, diluted in 3% BSA (1:200). Cells were counterstained with 10 μg/ml Hoechst 33342 (Sigma-Aldrich, MO, United States) for 15 min before being mounted onto microscope slides with fluorescent mounting medium (Dako, Agilent Technologies, CA, United States). Images were acquired on Carl Zeiss laser scanning confocal microscope (LSM) 780 equipped with the ELYRA PS1 super-resolution and PALM/STORM platform (Carl Zeiss, Germany) using Zen Black imaging software (2012).

For dSTORM imaging, cells were fixed for a second time in 4% v/v FA for 10 min after secondary antibody incubation to stabilise fluorophore labelling and thereafter incubated in 100 mM PBS-G (0.75 g glycine in 100 ml PBS-A) for 2 min to terminate fixation. Subsequently, cells were washed three times with 1x PBS-A and stored in PBS-A until image acquisition.

#### 2.1.6 Fluorescence Microscopy Imaging

##### 2.1.6.1 Confocal Laser Scanning Microscopy

Following treatments, cells transfected with mRFP-GFP-LC3 were counterstained with Hoechst and imaged using Plan-Apochromat 63x/1.4 oil immersion objective. 8–10 z-slices were acquired at intervals of 0.60 μm. Hoechst, GFP and mRFP were excited using lasers 405, 488 and 561 nm, respectively, with the emission light detected between 410–468 nm for Hoechst, 490–535 nm for GFP and 591–660 nm for mRFP. For quantification of punctate structures, z-stacks were projected as maximum intensity using the Zeiss Zen Black Software (2012). Images were exported and analysed for the number of autophagosomes (nA, yellow puncta) and autolysosomes (nAL, red puncta) in Fiji analysis software (Schindelin et al., 2012) using a “click count” function.

##### 2.1.6.2 Super Resolution-Structured Illumination Microscopy

Cells labelled for acetylated-α tubulin, α/(β)- tubulin and Hoechst were imaged using SR-SIM on the Elyra PS1 platform (Carl Zeiss, Germany). Images were acquired using 100x/1.46 Alpha Plan-Apochromatic oil immersion objective at 5 phase shifts and 3 rotations of the illumination grid per z-stack (0.50 μm interval). Image acquisition settings were set as follows; Hoechst: 405 nm excitation laser and BP 420–480 + LP 750 emission filter, acetylated-α tubulin: 488 nm excitation laser and BP 495–550 + LP 750 emission filter and α/(β)- tubulin: 561 nm excitation laser and BP 570–620 + LP 750 emission filter. For channel alignment, a slide containing multi-coloured 40 nm fluorescent beads was imaged under the same acquisition settings and used for an affine alignment of channels using the ZEN Black Elyra edition software (Carl Zeiss Microscopy).

##### 2.1.6.3 Direct Stochastic Optical Reconstruction Microscopy

Single molecule microscopy of cells labelled for APP and acetylated-α tubulin was performed. Molecules were activated using super-resolution Abbelight buffer (a kind gift from Pierre Bauër, Abbelight) or 1 M MEA buffer (#M9768, Sigma Aldrich). Blinking events were acquired using 100x/1.46 Alpha Plan-Apochromatic oil immersion objective using a 561 nm laser and fluorescence was detected with the EM-CCD iXon DU 897 camera. Imaging was performed in TIRF-uHP mode, 100% laser power, camera integration of 33 ms and EM gain of 150 to bleach the sample and force fluorophores to enter the dark state. Laser power was reduced to 2% while recording the events. A total of 50,000 events were recorded per cell. Raw data images were processed using a ZEN Black software where images were corrected for drifting and outliers. For APP analysis, images were further processed using FIJI analysis software. APP clusters were segmented using the Li thresholding algorithm (Li and Tam, 1998), and sizes were measured using the “analyze particle” function (Schindelin et al., 2012).

#### 2.1.7 Transmission Electron Microscopy

Following treatments cells were detached from the culture flask using Trypsin/EDTA. Thereafter, cells were washed with 1x PBS and fixed overnight at 4°C in 2.5% v/v glutaraldehyde (GA) in 0.1 M phosphate buffer (PB). Subsequently, cells were washed three times for 5 min with 0.1 M PB and embedded in 2% low melting agarose (Merck, SA) on ice. Small pieces (0.2 × 0.2 mm) of solidified agarose were post-fixed in 1% osmium tetroxide (SPI) in 0.1 M PB for 1 h and then washed for 5 min in 0.1 M PB followed by washing twice in dH2O. The samples were placed in sample baskets (3 wells) and underwent a process of dehydration and substitution using an automated tissue processor (Leica Biosystems), using the following conditions: 30 min in 2% uranyl acetate in 70% ethanol, 2 × 5 min in 70% ethanol, 5 min in 90% ethanol, 10 min in 2% uranyl nitrate in 96% ethanol, 3 × 10 min in 100% ethanol, 90 min in 1:1 Spurrs resin: 100% ethanol, and 2 × 1 h pure resin. After the cycle was complete, samples were embedded in Spurrs resin and capsules were polymerised at 60°C in the oven for 48 h. Ultra-thin sections, 150 nm thick were cut on a Leica EM UC7 ultramicrotome (Leica Microsystems, Germany), collected on 200 mesh copper grids (G200-Cu, Electron Microscopy Sciences) and stained with uranyl acetate and lead citrate. Grids were viewed in a JEOL JEM 1011 TEM (JEOL, Inc., Peabody, MA) operated at 120 kV and images were collected using a GATAN OneView camera.

Quantitative morphometric analysis was performed using Fiji software. Briefly, TEM images were exported as TIFF files and converted to 8-bit grayscale. After conversion, an outline was drawn using a drawing pen circulating autophagosomal and autolysosomal structures collectively known as autophagic vacuoles (AVs) (Eskelinen, 2008; Klionsky et al., 2016). Thereafter, images were thresholded using default settings; black and white (B & W) and dark background was used, and the grey values were set to 255 so that pixels with grey levels under a specified threshold were displayed as black, and those above as white pixels, thus demarcating the structures of interest against the background. The number and surface area (µm^2^) of AVs were analysed using the “analyze particle” function.

#### 2.1.8 3D Correlative Light and Electron Microscopy

##### 2.1.8.1 Super-resolution Structured Illumination Microscopy

For correlative imaging, GT1-7 cells transfected with a GFP-LC3-RFP-LC3ΔG DNA plasmid were seeded at 150,000 cells overnight onto 35-mm gridded glass-bottom dishes (#P35G-1.5-14-CGRD, MatTek Corporation, United States ). Cells were treated for 8 h with 1 and 10 μM Spd followed by treatment for 4 h in the presence and absence of 400 nM BAfA1. Thereafter, cells were fixed with 1:1 ratio of 8% v/v FA and culture medium for 15 min. Cells were rinsed 3x for 5 min and counterstained with 10 μg/ml Hoechst 33342 for 15 min. Thereafter, cells were rinsed 3x for 5 min and then imaged with SR-SIM. For each treatment group, cells were first imaged in the LSM mode with a low magnification (10x EC “Plan-Neofluar”) using a tile scan (4 × 4) to acquire a wide field of view that is inclusive of the grid. Thereafter, cells of interest were imaged at higher magnification (100x/1.46 Alpha Plan-Apochromatic oil immersion) in the SIM mode at 5 phase shifts and 3 rotations per z-stack (0.50 μm interval) to obtain final images to correlate with the electron microscopy images. Acquisition settings were set as follows; Hoechst: 405 nm excitation laser and BP 420–480 nm emission filter, GFP: 488 nm excitation laser and BP 495–550 + LP 750 emission filter and RFP: 561 nm excitation laser and BP 570–620 + LP 750 emission filter. For channel alignment, a slide containing multi-coloured 40 nm fluorescent beads was imaged under the same acquisition settings and used for an affine alignment of channels using the ZEN Black Elyra edition software (Carl Zeiss Microscopy).

##### 2.1.8.2 Focused Ion Beam Scanning Electron Microscopy

Following imaging with SR-SIM, as described above, cells were prepared for FIB-SEM imaging according to a published protocol (Russell et al., 2017). Briefly, cells were post-fixed in 2.5% (v/v) GA/4% (v/v) FA in 0.1 M PB for 30 min at RT. Subsequently, cells were washed in 0.1 M PB 5 × 3 min on ice, stained in 2% osmium tetroxide/1.5% potassium ferricyanide (v/v) for 60 min, on ice and washed in dH2O 5 × 3 min. Thereafter, cells were incubated in 1% thiocarbohydrazide in dH2O (w/v) for 20 min, washed in dH2O 5 × 3 min, before staining in 2% osmium tetroxide in dH2O (w/v) for 30 min. Following the staining procedure, cells were washed in dH2O for 5 × 3 min and incubated overnight in 1% aqueous uranyl acetate at 4°C. After overnight, cells were washed in dH2O, 5 × 3 min, stained with Walton’s lead aspartate for 30 min, at 60°C, washed in dH2O, 5 × 3 min. Coverslips were detached using a razor blade, dehydrated on ice, using a pre-chilled alcohol series in the following order: 20%, 50%, 70%, 90%, 100% EtOH, 5 min each, on ice, anhydrous 100% EtOH, 5 min, on ice, followed by 10 min at RT. Next, cells were incubated in 1:1 propylene oxide and Durcupan ACM^®^ (Sigma Aldrich, MO, United States ) resin mixture for 60 min, followed by incubation in Durcupan ACM^®^ for 90 min, twice and embedded in Durcupan ACM^®^ for 48 h at 60°C.

Following polymerization of the resin, coverslips were removed by brief immersion in liquid nitrogen. Resin embed samples were trimmed to the region of interest with reference to grid coordinates and prior light microscopy images. After thinning to <1 mm, the ROIs were attached to a standard 12.7 mm SEM stub using silver paint, sputter coated with a 5 nm layer of platinum, and mounted in the FIB-SEM (Zeiss Crossbeam 540 running with Atlas 5). The specific cells of interest were relocated by imaging through the platinum coating at an accelerating voltage of 20 kV and correlating to previously acquired fluorescence microscopy images. After preparation for milling and tracking, images were acquired at a voxel size of 5 × 5 × 5 nm throughout each region of interest using a 6–10 µs dwell time, depending on imaging stability and the final size of the selected imaging ROI. During acquisition, the SEM was operated at an accelerating voltage of 1.5 kV with 1 nA current. The EsB detector was used with a grid voltage of 1200 V. Ion beam milling was performed at an accelerating voltage of 30 kV and a current of 700 pA.

##### 2.1.8.3 Image Processing and Analysis

After initial registration (SIFT; Fiji, https://imagej.net/plugins/linear-stack-alignment-with-sift), the images were contrast normalised as required across the entire stack, and converted to 8-bit greyscale. The output images were batch processed to suppress noise, and to enhance sharpness and contrast (i. small radius gaussian blur; ii. smart sharpening with highlights suppressed, iii. application of grey levels adjustment as needed; Adobe Photoshop CC 2015). The level of processing was tailored to each individual dataset. If required, each dataset was rotated sequentially in XY and YZ to accurately match the light microscopy data (equivalent to XZ).

To identify autophagosomes with high precision, a correlation of SR-SIM and FIB-SEM datasets was performed. For 2D overlays, image stacks from SR-SIM were projected to maximum intensity projection using Zeiss Zen Black Software (2012) and manually aligned to a representative region of the FIB-SEM stack using Ec-CLEM plugin on Icy (Paul-Gilloteaux et al., 2017), with FIB-SEM image set as “target” and SR-SIM image as “source”. 20–30 matching landmarks were added within both datasets throughout the entire cell. The transformation was applied to the SR-SIM dataset to produce a 2D overlay and saved to be applied on another image in the FIB-SEM stack. Autophagosomes were identified from the 2D overlay, segmented manually from the FIB-SEM datasets and 3D reconstructions were made using 3dmod program of IMOD (Kremer et al., 1996). Briefly, the FIB-SEM stack was opened in IMOD and scaled to 5 nm pixel size. ROIs were located within the image stack based on the 2D overlay. Contours of each autophagosome were drawn manually on each slice through the entire volume. The “normal”, “sculpting” and “warp” drawing tools were used and a z-bridge of 5 was maintained. The “interpolate” function was used to complete the remaining contours. A new object was added for each new autophagosome. A total of 15–25 autophagosomes were segmented for each cell, the number of contours varied according to the size of the autophagosomes. Once segmentation had been completed, the model was opened in “model view” to display z-buffered wireframe rendering of model contour data with adjustable perspective. Each object was “capped” and “meshed” to form a solid 3D object.

For morphometric analysis [volume (µm^3^) and surface area (µm^2^)] of each autophagosome, binary masks. mrc files were created from imod segmentation files (.mod) by using the “imodmop-mask” command in the Linux (Ubuntu 20.04) command line. Prior to importing files into Amira (2019), binary masks .mrc files were down sampled and converted to .tiff. Binary masks were then used to create volumetric renders in Amira, and manually overlayed with the FIB-SEM data sets. For quantification of each vacuole, the “label measurements” option was applied to each segmentation set and exported as .csv files.

For movie production, the SR-SIM image stack was aligned to the FIB-SEM stack using Bigwarp plugin of the Fiji framework (Bogovic et al., 2016), after which the SR-SIM dataset was exported at the native resolution of the FIB-SEM dataset. A merged SR-SIM/FIB-SEM dataset was also created. For subsequent processing, each dataset was reduced in resolution from 5 to 20 nm^3^. Using Amira (2019.3, ThermoFisherSci), the warped SR-SIM, merged SR-SIM/FIB-SEM, and FIB-SEM datasets were visualised in combination with the model generated from segmentation in 3dmod and a movie was created and exported.

#### 2.1.9 Flow Cytometry

Flow cytometry was used to measure the production of mitochondrial reactive oxygen species (ROS) as well as cell death onset using MitoSox and Propidium Iodide. After treatment with low and a high concentration (1 and 10 μM) of spermidine for 8 h and followed by exposure to 3 mM PQ for 6 h, cells were washed gently in warm 1x PBS. Thereafter, cells were incubated with 5 μM MitoSOX Red or 1 μg/ml PI solution for 10 min at 37°C, protected from light. Carbonyl cyanide m-chlorophenyl hydrazone (CCCP) at 5 μM was used as a positive control. The fluorescence intensity of MitoSOX Red and PI were measured on the BD FACSAria IIu flow cytometer (BD Biosciences, CA, United States). A minimum of 20,000 gated cells were collected using a 488 nm laser and 610/20 bandpass filter. The mean fluorescence intensity was assessed from three independent experiments. Results were expressed as a percentage of controls.

### 2.2 *In Vivo* Study

#### 2.2.1 Animals

Eight-week-old male C57BL/6 mice expressing GFP-LC3 were used in this study. GFP-LC3 transgenic mice breeding pairs were purchased from Riken, Bio-Resource Centre in Japan (#BRC00806) and mice were bred at the breeding facility at Stellenbosch University according to the previously described protocol (Mizushima et al., 2004; Mizushima, 2009). 72 GFP-LC3 mice were randomly allocated into six treatment groups: control, PQ, 0.3 mM Spd, 0.3 mM + PQ, 3 mM Spd and 3 mM Spd + PQ, with a total of 12 mice (N = 12) per group. Mice were kept on a 12 h day/night cycle at a constant temperature of 22°C and 40% humidity. Mice were monitored and weighed for the duration of the study. All animals had free access to standard chow and drinking water *ad libitum*. All procedures for handling the mice in this study were reviewed and approved by the animal research ethics committee (SU-ACUD16-00175), at Stellenbosch University, South Africa.

#### 2.2.2 Treatment Conditions

Mice in the control, PQ, and the combination groups (0.3 mM + PQ and 3 mM Spd + PQ) were subjected to a total of 6 injections of saline solution or PQ solution given at 10 mg/kg which was administered intraperitoneally every 3 days for 3 weeks (Chen L. et al., 2012). Animals in the spermidine group and the combination groups received previously reported doses of 0.3 and 3 mM Spd in drinking water (LaRocca et al., 2013; Eisenberg et al., 2016) every day for 21 days. Spermidine (Sigma-Aldrich, St Louis, MO, United States) was prepared fresh in tap water every 3 days from a 1 M aqueous stock solution (spermidine/HCl pH 7.4) stored at −20°C, while PQ (Sigma-Aldrich, St Louis, MO, United States) was prepared fresh in saline solution before injection.

#### 2.2.3 Protein Extraction for Western Blot Analysis

Protein lysates were harvested from brain tissue (hippocampus and cerebral cortex) with RIPA buffer supplemented with protease and phosphatase inhibitors. In brief, brain tissue samples were minced in RIPA buffer using scissors while on ice, after which tissue samples were rapidly homogenised by sonication on ice, using 3 bursts of 5 s (Kine Matica Polytron PT2100 homogenizer, ThermoFischer Scientific). Protein content of the lysates was determined using the Direct Detect system and samples were prepared for western blotting as described above. Primary antibodies to anti-acetylated-α-tubulin 6-11B (#23950, Santa Cruz, 1:5000), anti-APP (#2452, Cell Signalling, 1:1000), anti- Sequestosome1/p62 (#ab56416, abcam, 1:5000), and 4-Hydroxy-2-nonenal (#ab46545, Abcam, 1:1000) as well as secondary Horse Radish Peroxidase linked antibodies anti-Rabbit IgG (#CST7074S, Cell Signalling, 1:5000) and anti-Mouse IgG (#CST7076S, Cell Signalling, 1:5000) were used.

### 2.3 Statistical Analysis

The results are expressed as mean values ±SEM and were analysed by one-way Analysis of Variance (ANOVA) with a Fischer LSD post hoc correction. Graph Pad Prism (v8) was employed to perform statistical tests. Data were considered statistically significant with a *p*-value < 0.05.

## 3 Results

### 3.1 Spermidine Induces Autophagic Flux and Improves Cellular Viability in a Concentration-Dependent Manner

**FIGURE 1:**
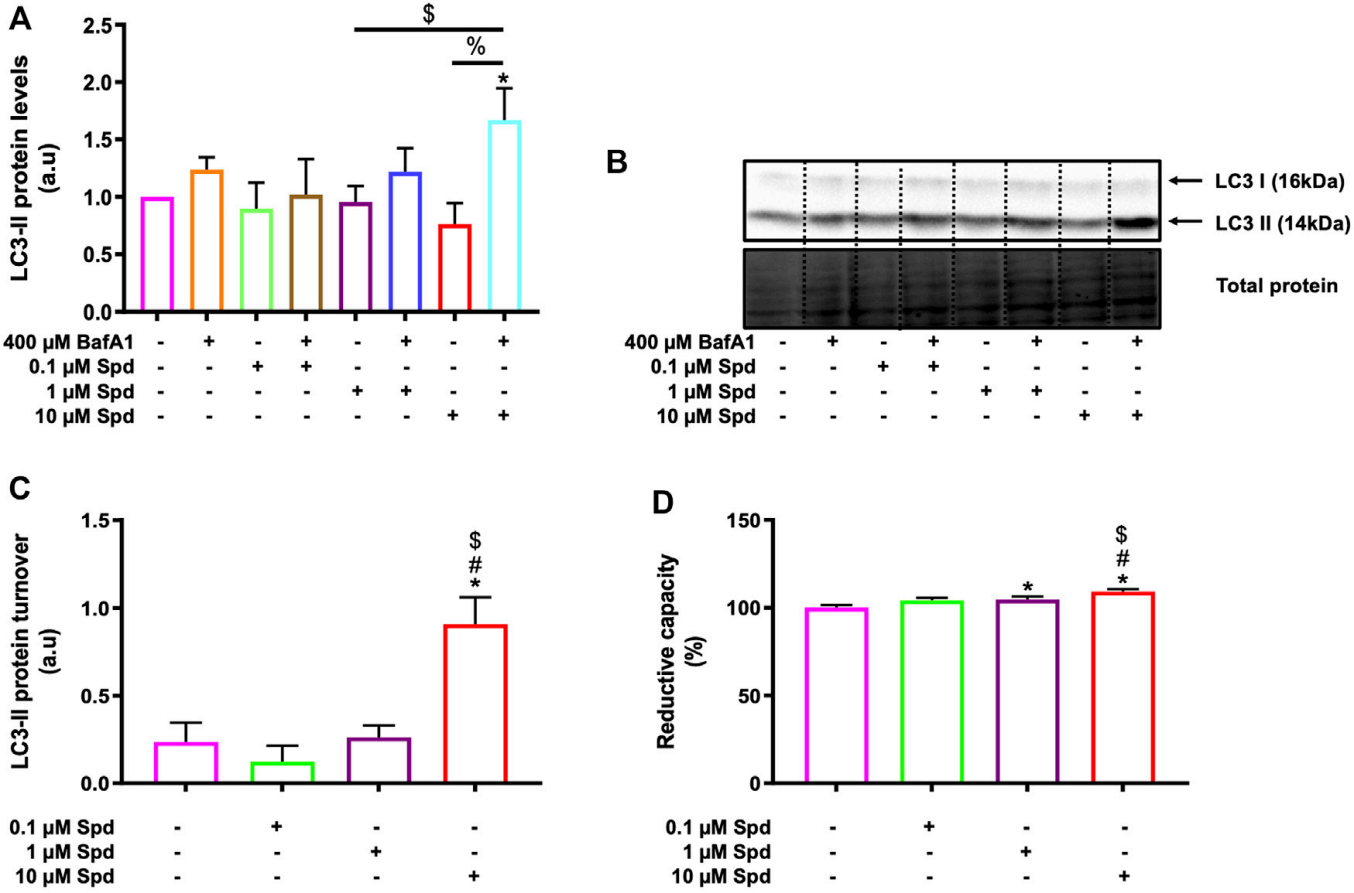
Spermidine induces autophagic flux and improves cellular viability in a concentration-dependent manner. **(A)** densitometric quantification and **(B)** representative western blot for LC3-II protein levels upon autophagy induction using three different concentrations (0.1, 1 and 10 μM) of spermidine in the presence and absence saturating concentrations of bafilomycin. Data are presented as mean ± SEM, *n* = 3. * = *p* < 0.05 vs. control, $ = *p* < 0.05 vs. 0.1 μM Spd + BafA1 and % = *p* < 0.05 vs. 10 μM Spd, arbitrary units (a.u). **(C)** LC3-II turnover revealing a concentration-dependent flux by spermidine. * = *p* < 0.05 vs. control, # = *p* < 0.05 vs. 0.1 μM Spd and $ = *p* < 0.05 vs. 1 μM Spd. **(D)** Reductive capacity in GT1-7 cells following treatment with three different concentrations of spermidine. Data are presented as mean ± SEM, *n* = 3 with 6 replicates per group. * = *p* < 0.05 vs. control, # = *p* < 0.05 vs. 0.1 μM Spd and $ = *p* < 0.05 vs. 1 μM Spd.

Three different concentrations of spermidine were tested in the presence and absence of BafA1 to determine a low and high concentration that induces autophagic flux without causing cellular death in GT1-7 cells. A WST-1 assay and western blotting were used to measure cellular viability and endogenous levels of LC3-II (microtubule-associated protein 1 light chain) respectively. LC3-II is a key component of mature autophagosomes (Kabeya et al., 2000) that is widely used to assess autophagosome abundance. LC3-II levels were significantly increased in the 10 μM Spd + BafA1 treated group compared to the control (untreated) group and 0.1 μM Spd + BafA1 treated group (Figures 1A,B). To reveal the effect of spermidine on LC3-II levels better, we assessed LC3-II turnover determined by subtracting mean values of BafA1 treated groups (BafA1, 0.1 μM Spd + BafA1, 1 μM Spd + Baf A1 and 10 μM Spd + BafA1) in Figure 1A from BafA1 untreated groups (control, 0.1 μM Spd, 1 μM Spd and 10 μM Spd), respectively. Spermidine at 10 μM showed a significant increase in LC3-II levels compared to the control, 0.1 and 1 μM Spd treated groups (Figure 1C). The WST-1 assay revealed that spermidine at 1 and 10 μM improved cellular viability compared to the control group (Figure 1D). Based on these results spermidine at 1 and 10 μM were used for subsequent experiments.

### 3.2 Spermidine Induces Autophagic Flux in a Concentration-dependent Manner Which Is Most Sensitively Revealed Through Vesicle-Based Analysis

**FIGURE 2:**
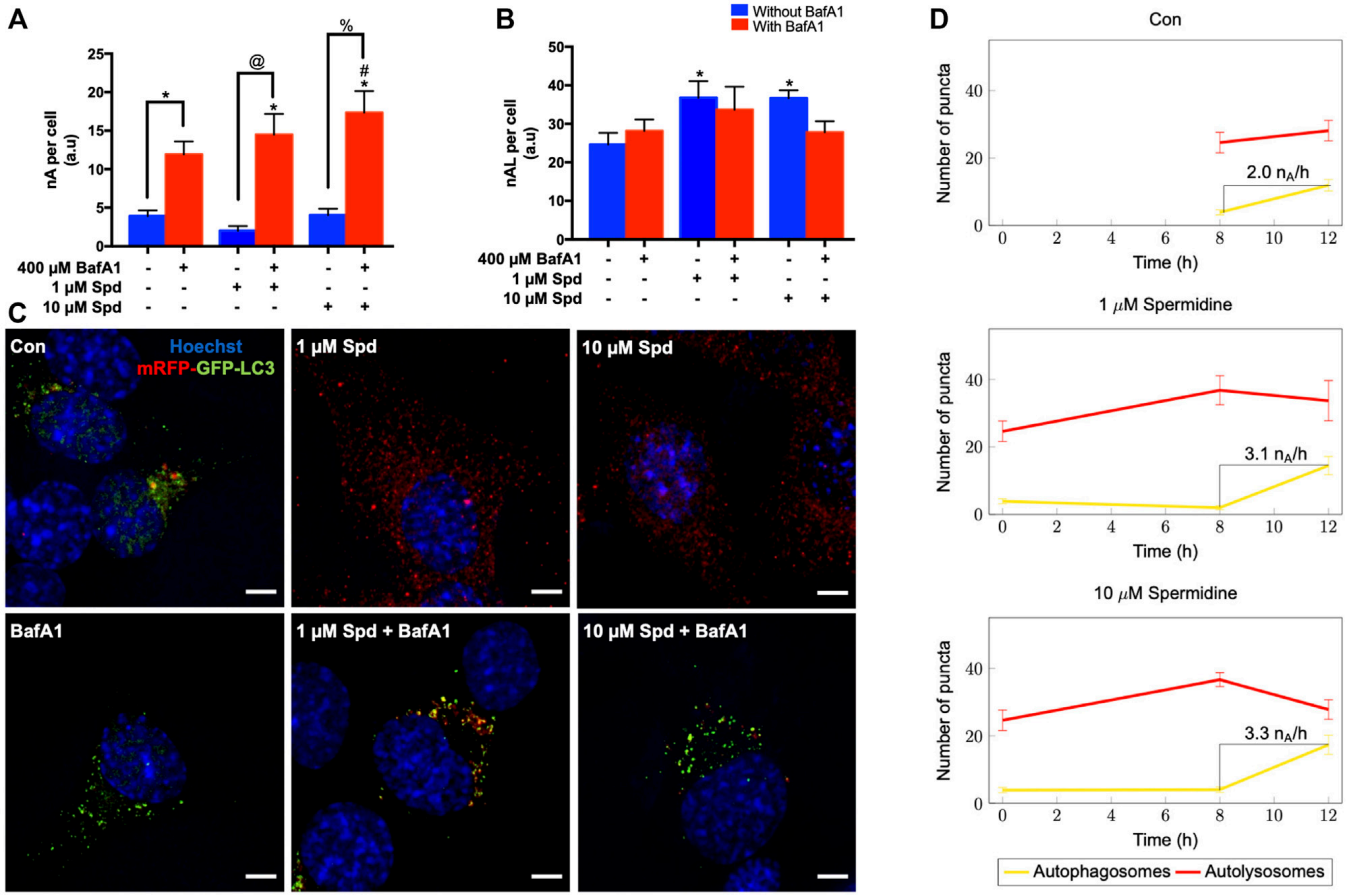
Spermidine induces autophagic flux in a concentration-dependent manner. **(A,B)** quantitative analysis and **(C)** representative fluorescence micrographs of autophagosome pool size **(A,C)** and autolysosome pool size **(B,C)** in GT1-7 cells following treatment with 1 and 10 μM spermidine in the presence and absence of saturating concentrations bafilomycin. Data are presented as mean ± SEM, n = 3, with a total of 30 cells analysed per treatment group. * = *p* < 0.05 vs control, # = *p* < 0.05 vs BafA1, @ = *p* < 0.05 vs 1 μM Spd and % = *p* < 0.05 vs 10 μM Spd, arbitrary units (a.u). Scale bar: 5 μm. **(D)** Autophagosome flux under basal and induced conditions calculated from the initial slope of autophagosomes accumulation. Autophagosome flux increased in a concentration-depended manner, raising from 2.0 autophagosomes/h/cell under basal levels to 3.1 autophagosomes/h/cell and 3.3 autophagosomes/h/cell under induced conditions with 1 and 10 μM Spd respectively.

To further characterise the autophagic flux profile of 1 and 10 μM Spd, fluorescence and TEM-based analysis was performed in GT1-7 cells in the presence and absence of BafA1. For fluorescence-based analysis, GT1-7 cells expressing mRFP-GFP-LC3 were used to determine the number of autophagosomes (nA, yellow puncta) and autolysosomes (nAL, red puncta). In addition, autophagosome flux (J), which is the initial rate of increase in nA per cell after treatment with BafA1 and the transition time (τ) required for a cell to turnover its entire autophagosome pool were determined (Loos et al., 2014; du Toit et al., 2018a; du Toit et al., 2018b). mRFP-GFP-LC3 is a pH-sensitive tandem fluorescent probe that fluoresces red (mRFP) in the autolysosome due to the quenching of GFP signal because of the auto-lysosomal acidity, while fluorescing red (mRFP) and green (GFP) in the autophagosome resulting in a yellow signal (Yoshii and Mizushima, 2017). As such, when autophagic flux is increased, both yellow puncta (mRFP and GFP) and red puncta (mRFP only) are increased. Inhibition of autophagosome fusion with lysosomes results in an increase in the number of yellow puncta and a concurrent decrease in red puncta (Yoshii and Mizushima, 2017). Our results showed that 1 μM Spd + BafA1, 10 μM Spd + BafA1 and BafA1 treated groups significantly increased the number of autophagosomes compared to the control group (Figures 2A,C), while 1 and 10 μM Spd treatment significantly increased the number of autolysosomes (Figures 2B,C). Interestingly, only 10 μM Spd + BafA1 group enhanced the accumulation of autophagosomes compared to BafA1 treated group, suggesting a concentration-dependent increase in autophagic flux. Furthermore, 1 μM Spd + BafA1 and 10 μM Spd + BafA1 significantly increased autophagosome accumulation compared to 1 μM Spd and 10 μM Spd, respectively. Autophagosome flux was enhanced in a concentration-dependent manner, raising from 2.01 nA/h/cell under basal levels to 3.1 nA/h/cell and 3.3 nA/h/cell under induced conditions using 1 and 10 μM Spd respectively (Figure 2D). Analysis of the transition time (τ) showed that 1 μM Spd treated cells required 0.64 h to turn over their autophagosome pool, while 10 μM Spd required 1.21 h compared to 2 h needed by the control cells to turn over its pool.

**FIGURE 3:**
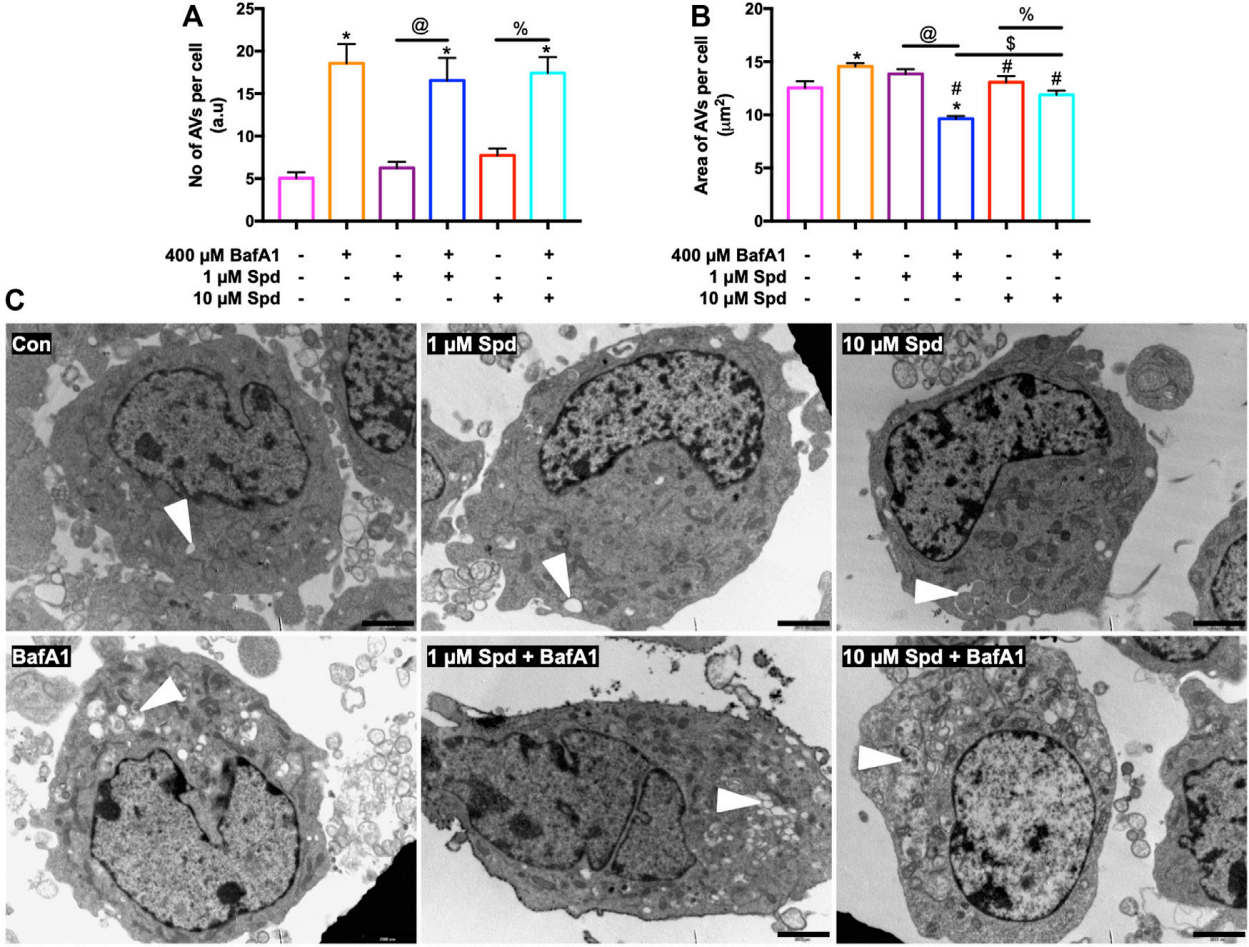
Spermidine affects the size of AVs in a concentration-dependent manner but has no effect on the number of AVs. **(A,B)** morphometric analysis and **(C)** representative TEM micrographs of autophagic vacuoles (AVs) in GT1-7 cells following treatment with 1 and 10 μM spermidine in the presence and absence of saturating concentrations bafilomycin. Data are presented as mean ± SEM, *n* = 2 with a total of 30 images analysed per treatment group. * = *p* < 0.05 vs control, # = *p* < 0.05 vs. BafA1, $ = *p* < 0.05 vs. 1 μM Spd + BafA1, @ = *p* < 0.05 vs. 1 μM Spd and % = *p* < 0.05 vs. 10 μM Spd, arbitrary units (a.u). Arrowheads indicate AVs. Scale bar: 2000 nm.

Morphometric analysis with TEM revealed that BafA1, 1 μM Spd + BafA1 and 10 μM Spd + BafA1 treated groups significantly increased the number of AVs compared to the control group (Figures 3A,C). No significant differences were observed in the 1 μM Spd + BafA1 and 10 μM Spd + BafA1 treated groups compared to BafA1 treated group (Figures 3A,C). Surface area was significantly reduced in 1 μM Spd + BafA1 compared to 1 μM Spd and in 10 μM Spd + BafA1 compared 10 μM Spd (Figures 3B,C). Importantly, 1 μM Spd + BafA1 reduced the size of AVs compared to 10 μM Spd + BafA1, suggesting a concentration-dependent effect.

### 3.3 Spermidine Induces Autophagosomal Recruitment of LC3-GFP, Supporting Autophagy Induction

**FIGURE 4:**
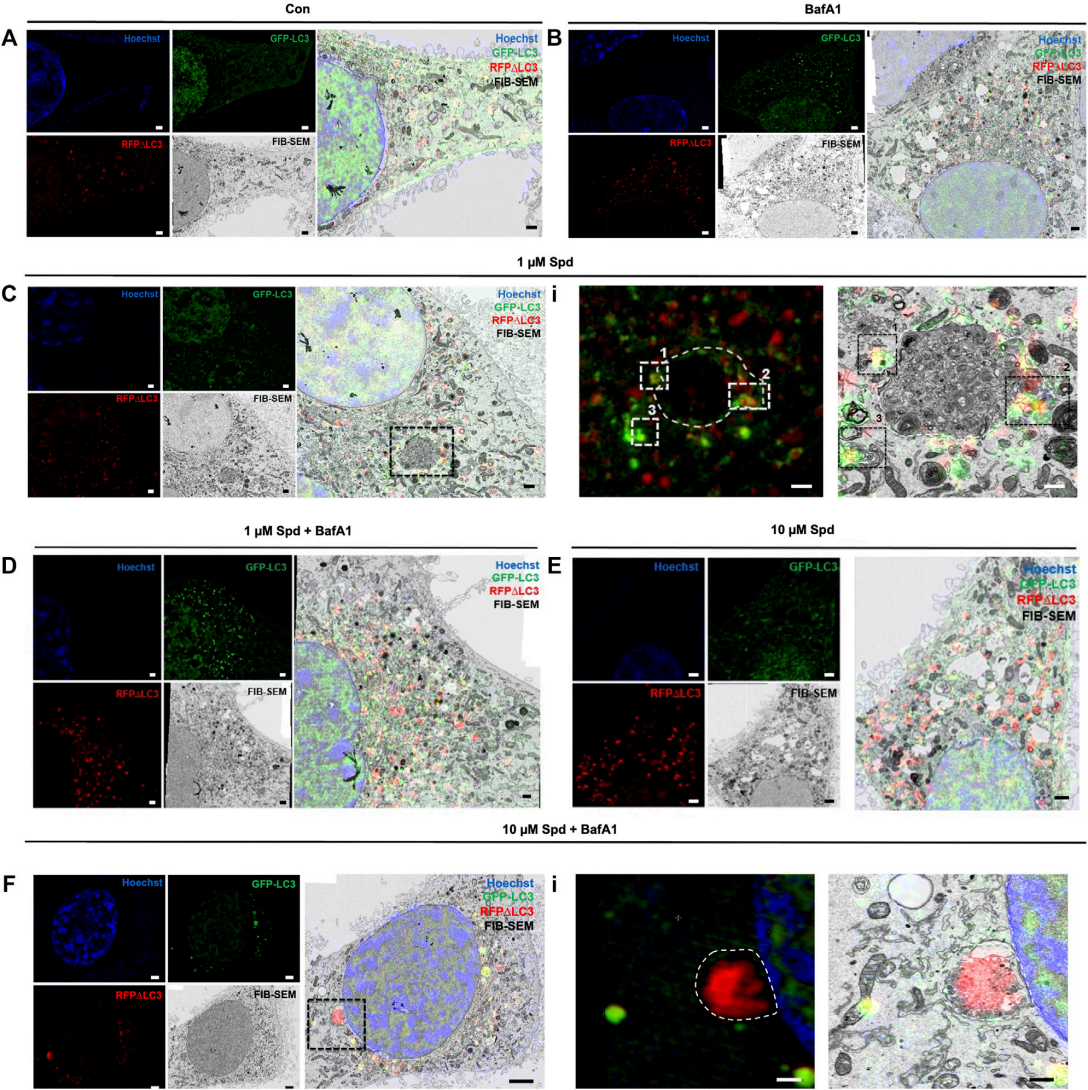
Spermidine induces autophagy. **(A–F)** Three colour micrograph of SR-SIM, FIB-SEM and overlay of SR-SIM and FIB-SEM showing the accumulation of GFP-LC3 and RFP-LC3ΔG positive structures following treatments. **(C**,**F)** complex structures of high electron density. **(Ci)** region of interest showing GFP-LC3 and RFP-LC3ΔG positive structures decorating conglomerate structure. (**Fi**) region of interest showing the localisation of RFP-LC3ΔG positive structures inside the conglomerate structure as part of cytoplasmic cargo. Scale bar: 1 μm.

**FIGURE 5:**
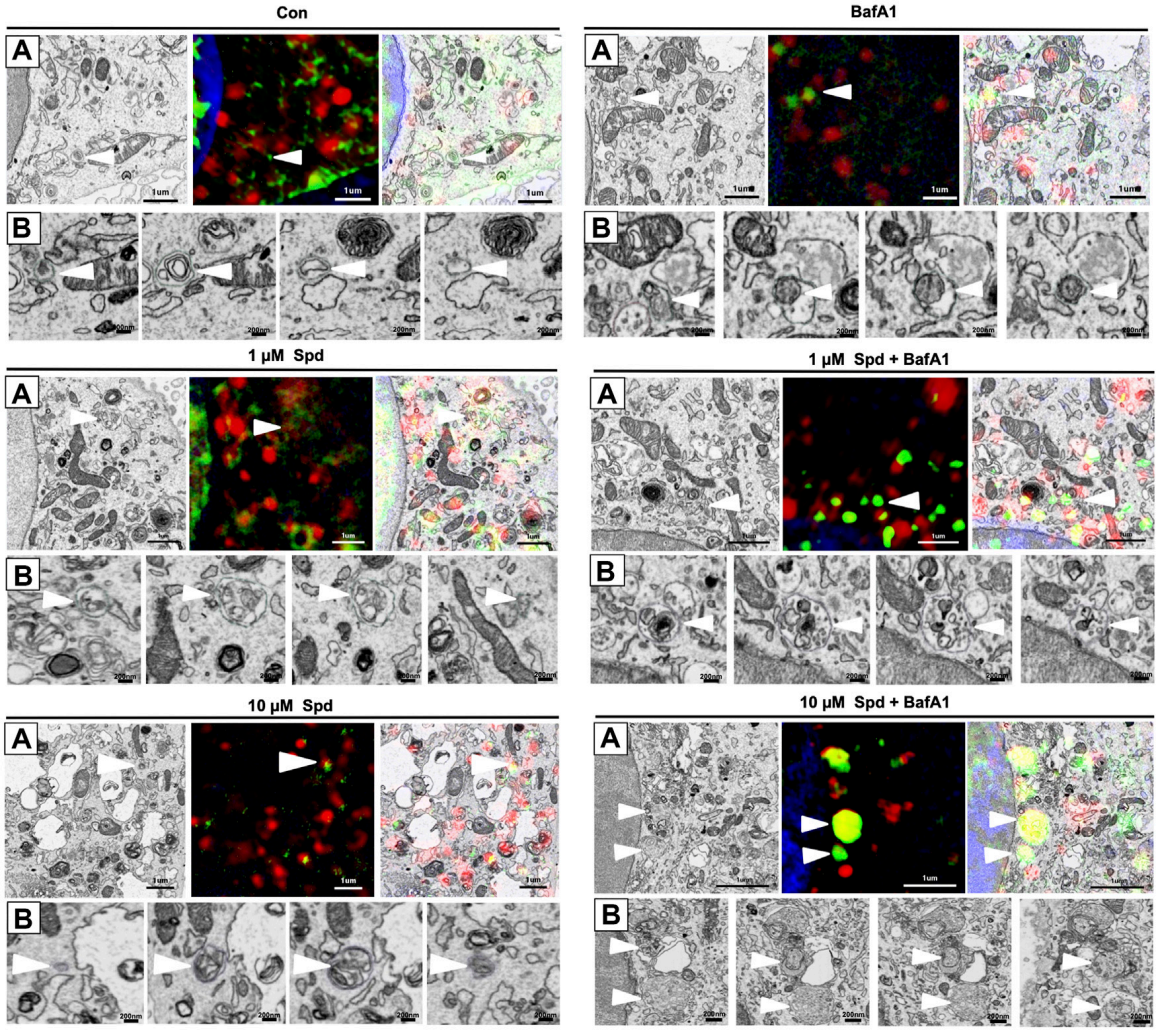
Differential response in cargo selection upon treatment. **(A)** Selective region of interest indicating autophagosome (white arrow heads) in FIB-SEM (Left), SR-SIM (middle) and CLEM (right) for the control (untreated) group, 1 μM Spd, 1 μM Spd + BafA1, 10 μM Spd and 10 μM Spd + BafA1. **(B)** FIB-SEM micrograph showing distinct cargo contained within the autophagosome (white arrow heads) following treatments. 10 μM Spd + BafA1 treated group recruits cargo characterised by the abundance of small intra-autophagosomal vesicle-like structures of homogeneous electron density. Scale bar: 1 μm and 200 nm.

To identify and localise GFP-LC3 positive structures i.e., autophagosomes, with high precision in the context of EM ultrastructure, and to monitor autophagic flux in the context of autophagosome formation, 3D-CLEM was performed using GT1-7 cells expressing GFP-LC3-RFP-LC3ΔG. This third-generation autophagy plasmid is a recently developed fluorescence probe that allows to measure and directly visualise autophagy flux (Kaizuka et al., 2016). When expressed in cells, it is cleaved by ATG4 into equimolar amounts of GFP-LC3 and RFP-LC3ΔG, where GFP-LC3 binds to the inner and outer membrane of the autophagosome and therefore is degraded during autophagy, while RFP-LC3ΔG remains in the cytosol, thus serving as an internal control (Kaizuka et al., 2016). Autophagic flux can hence be estimated by monitoring a decrease in GFP fluorescence compared to RFP fluorescence. 3D CLEM analysis using SR-SIM and FIB-SEM (Figure 4) allowed high-resolution imaging where autophagosomes could be identified with high precision. Upon inspection of SR-SIM micrographs, an increase in RFP-LC3ΔG positive structures was observed in the 1 μM Spd (Figure 4C; Supplementary Figure S1), 10 μM Spd (Figure 4E; Supplementary Figure S1) treated group compared to the control (untreated) group (Figure 4A; Supplementary Figure S1), suggesting heightened autophagic flux. GFP-LC3 positive structures were increased in the BafA1 (Figure 4B; Supplementary Figure S1), 1 μM Spd + BafA1 (Figure 4D; Supplementary Figure S1), 10 μM Spd + BafA1 (Figure 4F; Supplementary Figure S1) treated groups compared to control (untreated), 1 μM Spd and 10 μM Spd treated groups, respectively. Importantly, GFP-LC3 positive structures increased in abundance in the 1 μM Spd + BafA1 and 10 μM Spd + BafA1 treated group compared to the BafA1 treated group, suggesting an induced flux. Ultra-structural analysis with CLEM revealed the presence of complex structures in the 1 μM Spd and 10 μM Spd + BafA1 treated group that were GFP-LC3 and RFP-LC3ΔG positive (Figures 4Ci; Supplementary Video S1 and Figures 4Fi), or only RFP-LC3ΔG positive (Figures 4Fi), localizing in vacuolar structures of high electron density, either decorating the conglomerate structure (Figures 4Ci; Supplementary Video S1) or forming part of the cytoplasmic cargo (Figures 4Ci; Supplementary Video S1; Figures 4Fi). GFP-LC3 positive structures appeared to be localised in vacuolar structures of relatively low electron density (Figure 5). However, the cargo material inside the vacuolar structures was distinct in each treatment, with 10 μM Spd + BafA1 treated cell presenting cargo characterised by the abundance of small intra-autophagosomal vesicle-like structures of homogeneous electron density.

### 3.4 The Effect of Spermidine on Cargo Clearance and Size Regulation

**FIGURE 6:**
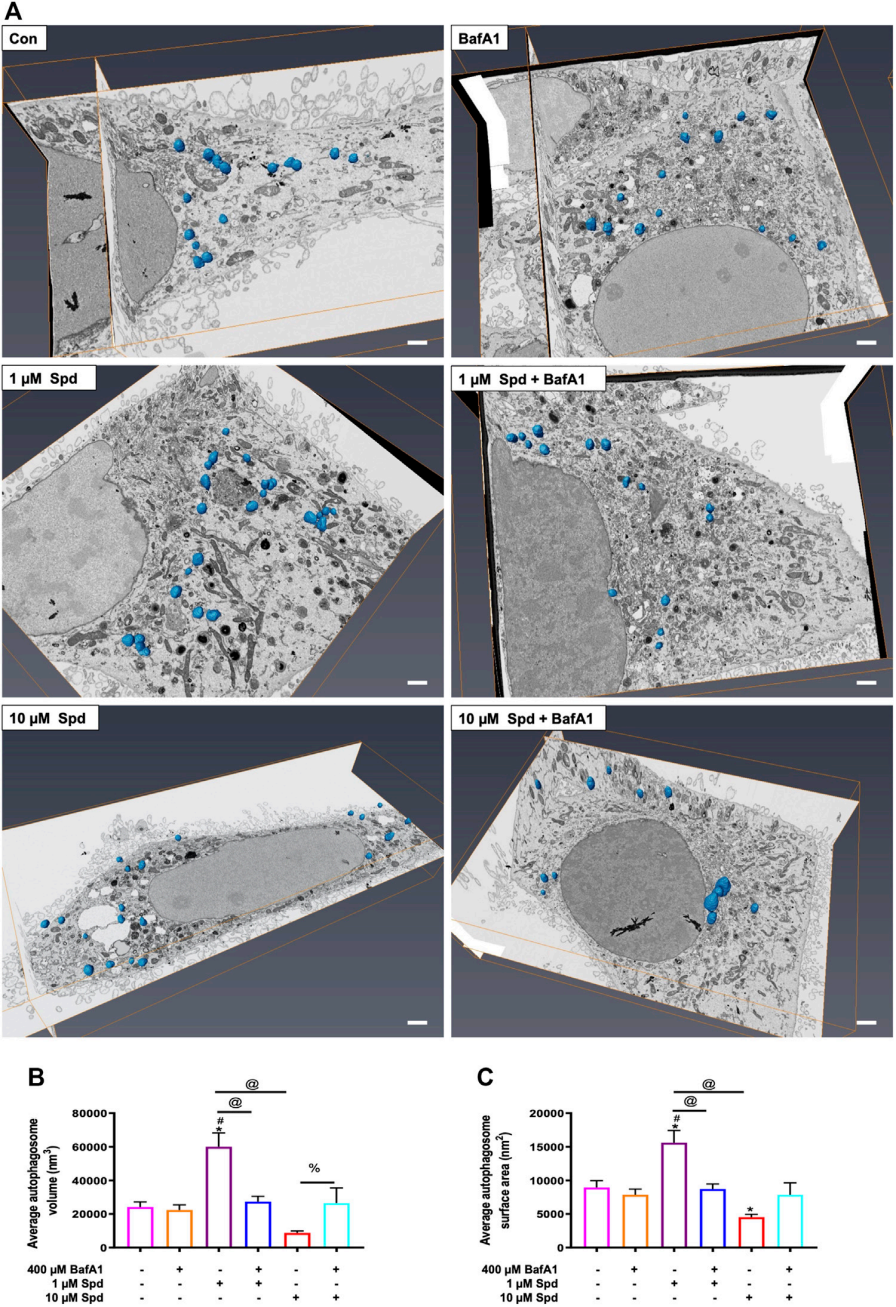
Effect of spermidine on cargo clearance and size regulation. **(A)** Orthogonal views of FIB-SEM stack showing segmented autophagosomes (scale bar: 1 μm). **(B)** Morphometric analysis of autophagosome volume and **(C)** surface area following treatments. Data are presented as mean ± SEM. A total of 15–25 autophagosomes analysed per treatment group. Spermidine at low concentration induces autophagosomes capable of large volume clearance. * = *p* < 0.05 vs. control, # = *p* < 0.05 vs. BafA1, @ = *p* < 0.05 vs. 1 μM Spd and % = *p* < 0.05 vs. 10 μM Spd.

Taking advantage of the 3-dimensional data of both SR-SIM and FIB-SEM, we assessed whether 1 and 10 μM of spermidine influenced the surface area and volume of autophagosomes, providing a possible indication of size regulation but also the capacity of proteinaceous cargo clearance. Hence, autophagosomes were manually segmented through the entire volume of the cell to generate a three-dimensional model, which showcases their localization within the entire volume of the cell (Figure 6A; Supplementary Figure S3). Morphometric analysis revealed that treatment with 1 μM Spd significantly increased the volume of autophagosomes compared to the control (untreated), BafA1, 1 μM Spd + BafA1 and 10 μM Spd treatment group (Figure 6A,B; Supplementary Video S2, Supplementary Figure S3). Similarly, autophagosome surface area was significantly increased in the 1 μM Spd group compared to the control (untreated), BafA1, 1 μM Spd + BafA1 and 10 μM Spd treatment group (Figure 6A,C; Supplementary Figure S3), while the surface area was significantly reduced in the 10 μM Spd treated group compared to the control (untreated) group.

### 3.5 Low Concentration of Spermidine Improves Cellular Viability, Reduces ROS Production, and Protects Against Cell Death Onset in Paraquat Treated Cells

**FIGURE 7:**
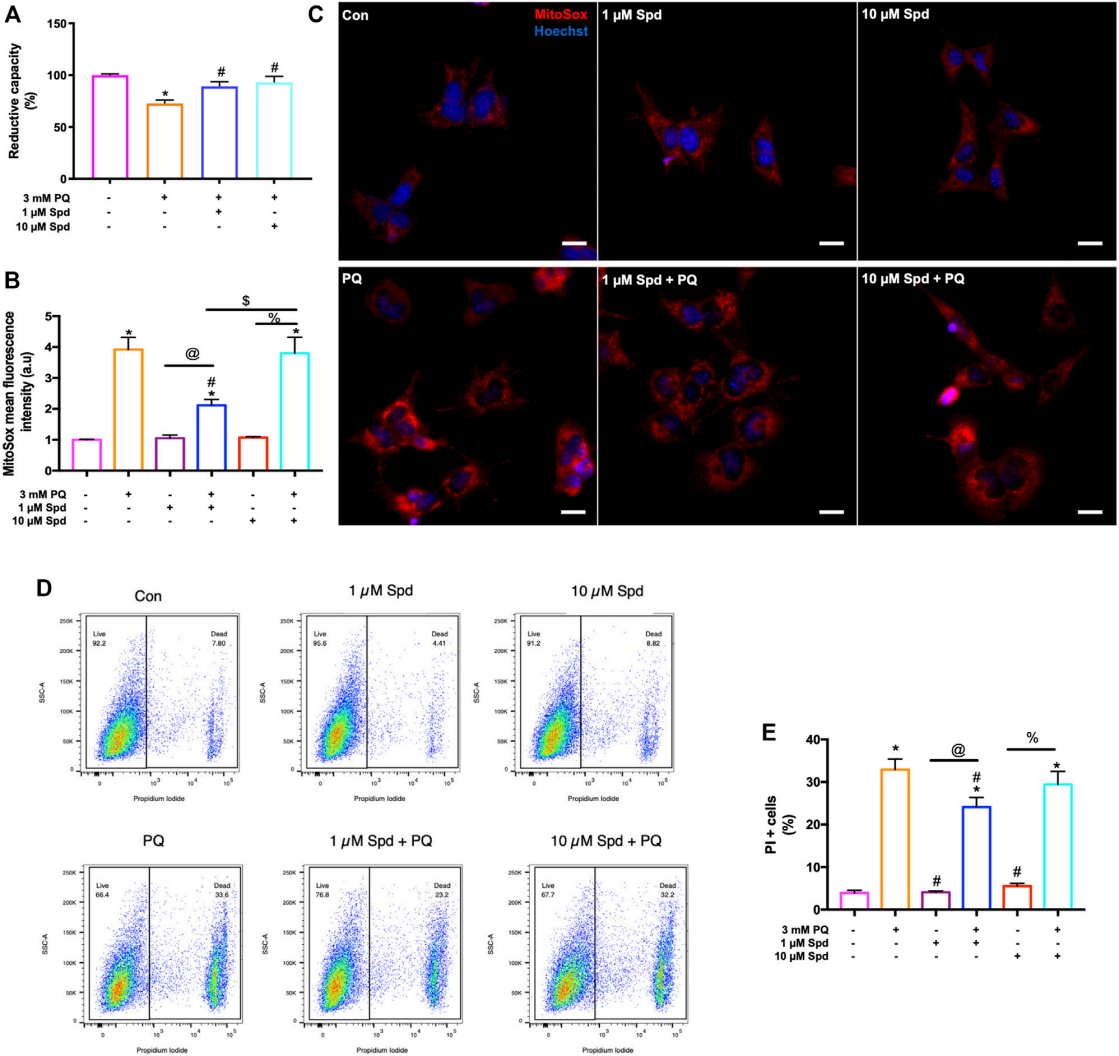
Low concentration of spermidine reduces ROS production and delay cell death onset following paraquat-induced neurotoxicity. **(A)** Reductive capacity indicating an improvement in cellular viability in cells pre-treated with spermidine compared to PQ alone. Data are presented as mean ± SEM, *n* = 3 with 6 replicates per group. * = *p* < 0.05 vs control, # = *p* < 0.05 vs PQ. **(B)** quantitative analysis of mitochondrial ROS production and **(C)** representative fluorescence micrographs following treatments. MitoSox fluorescence intensity was increased in PQ treated group, while decreased with 1 μM Spd + PQ. Scale bar: 20 μm. **(D,E)** quantitative analysis of cell death onset following treatments. PI positive cells were reduced in 1 μM Spd + PQ compared to PQ treated group. Data are presented as mean ± SEM, *n* = 3 with 2 replicates per group. * = *p* < 0.05 vs control group, # = *p* < 0.05 vs. PQ, @ = *p* < 0.05 vs. 1 μM Spd, % = *p* < 0.05 vs. 10 μM Spd, and $ = *p* < 0.05 vs 1 μM Spd + PQ.

To assess concentration-dependent effects of spermidine, GT1-7 cells treated with 1 & 10 μM Spd followed by exposure to 3 mM PQ were assessed for cellular viability, ROS production and loss of membrane integrity [propidium iodide (PI) uptake] using a WST-1 assay and flow cytometry. Analysis showed that PQ treated groups displayed a significantly reduced cellular viability, while both 1 μM Spd + PQ and 10 μM Spd + PQ significantly improved cellular viability (Figure 7A). Flow cytometry and immunofluorescence analysis of ROS production revealed a significant increase in the PQ treated group (Figures 7B,C), whereas 1 μM Spd + PQ significantly decreased ROS production. PI positive cells were significantly increased in the PQ treated group while decreased in the 1 μM Spd + PQ treated group (Figures 7D,E).

### 3.6 Low Concentration of Spermidine Increases the Acetylation of *α*-tubulins in Paraquat Treated Cells

**FIGURE 8:**
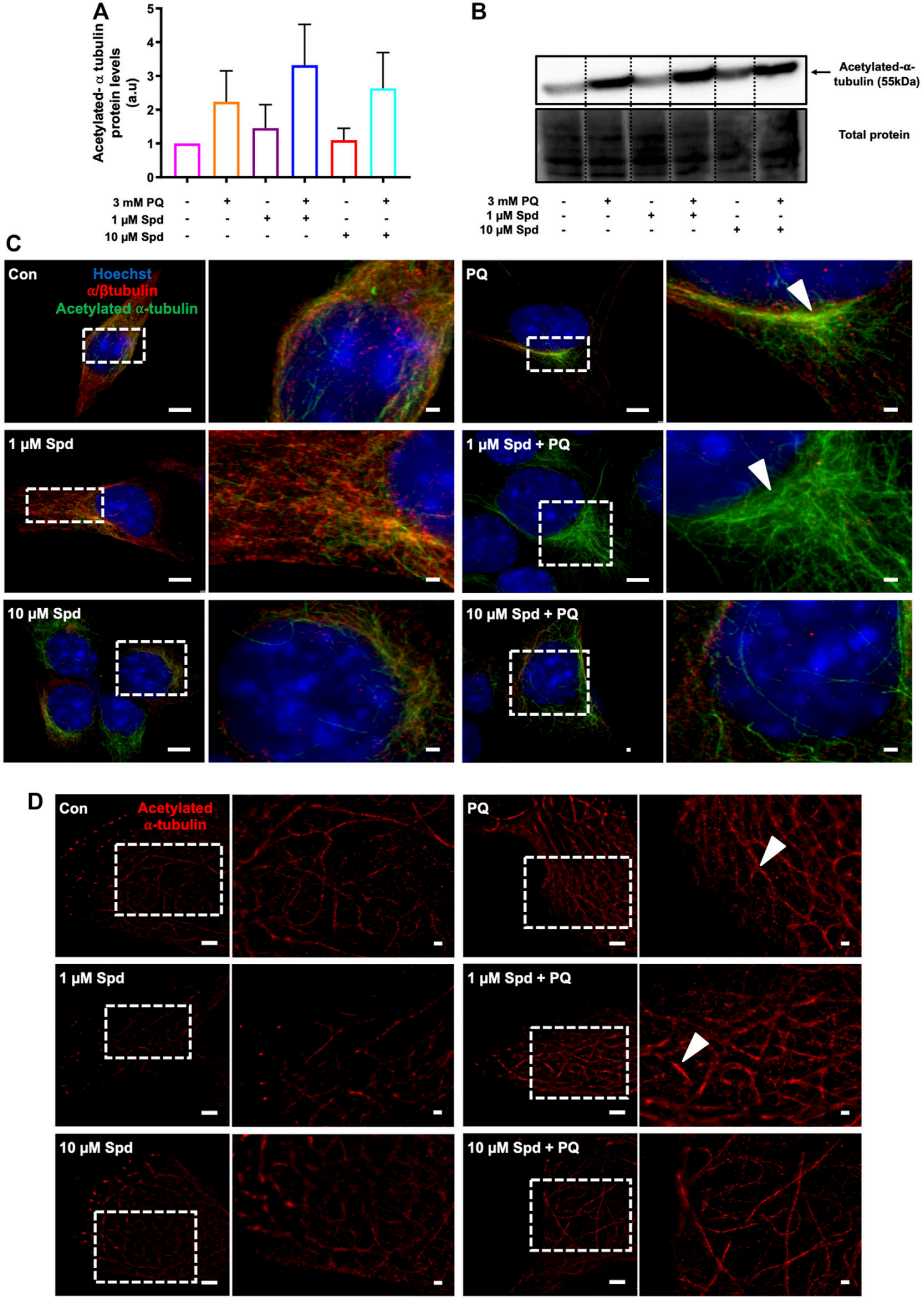
Spermidine increases the acetylation signal of tubulin in paraquat treated cells. **(A)** densitometric quantification and **(B)** representative western blot for acetylated α-tubulin protein levels in GT1-7 cells following treatment with a low and high concentration of spermidine followed by exposure to paraquat. Data are presented as mean ± SEM, *n* = 3. **(C,D)** representative micrographs for acetylated α-tubulin with SR-SIM (scale bar: 5 and 1 μm) and dSTORM (scale bar: 2 and 0.5 μm) showing highest intensity signal with 1 μM Spd + PQ compared to PQ. Arrowheads indicate regions of strong acetylated α-tubulin signal.

Autophagosome transport relies on a functional tubulin network (Xie et al., 2010; Jahreiss et al., 2008) with the autophagy machinery closely engaging with the cell’s acetylation processes (Pietrocola et al., 2015). Here we assessed the effect of a low and of a high concentration of spermidine on tubulin acetylation following PQ treatment. Western blot analysis revealed no significant differences in the levels of acetylated α-tubulin between treatment groups (Figures 8A,B), although a trend towards an increase was observed in the 1 μM Spd + PQ treated group compared to PQ alone. Upon visual inspection of the fluorescence micrographs from SR-SIM and single-molecule microscopy, acetylated α-tubulin signal and density was indeed enhanced in cells of the 1 μM Spd + PQ treated group compared to PQ alone (Figures 8C,D).

### 3.7 Spermidine Improves Cellular Viability and Reduces the Number of APP Clusters By Enhancing Autophagy in N2aSwe Cells

**FIGURE 9:**
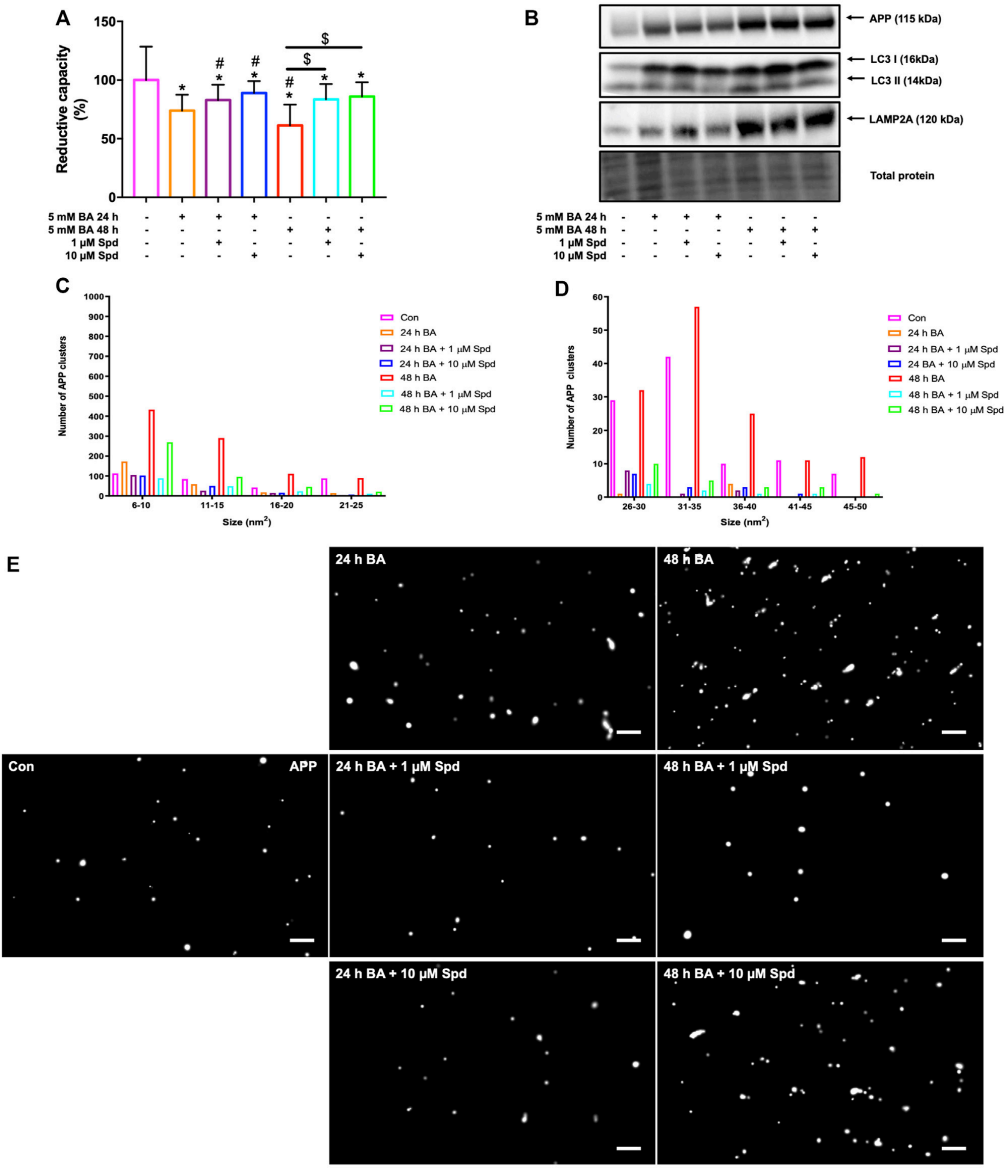
Spermidine protects against APP-induced cytotoxicity and reduces the size and number of APP clusters. **(A)** Reductive capacity and **(B)** western blot analysis of APP, LC3-I/II and LAMP2A protein levels following treatment with a low and high concentration of spermidine in N2aSwe cells treated with BA overtime to overexpress APP (*n* = 3). * = *p* < 0.05 vs. control, # = *p* < 0.05 vs. 24 h BA and $ = *p* < 0.05 vs. 48 h BA. **(C–E)** Size distribution of APP clusters and representative dSTORM micrographs in Gauss mode showing the localisation of APP clusters. Spermidine reduces the size and number of APP clusters. Scale bar: 0.5 μm.

We next evaluated the protective effects of a low and of a high concentration of spermidine in N2aSwe cells overexpressing APP. A time-dependent decrease in cellular viability was observed following APP overexpression for 24 and 48 h, while spermidine treatment significantly improved cellular viability at both time points (Figure 9A). In addition, N2aSwe cells exhibited a time-dependent increase in APP, LC3-II and LAMP2A protein levels (Figure 9B). dSTORM analysis revealed a size distribution profile of APP clusters ranging from 6 to 50 nm^2^ (Figure 9C–E; Supplementary Figure S2). Treatment with BA for 48 h resulted in an increase in the number of APP clusters of all sizes compared to the control, while BA for 24 h displayed a higher number of APP clusters at sizes 6–10 nm^2^. In addition, spermidine treatment after 24 h BA resulted in the clearance of APP clusters as evident by the reduction in the number of APP clusters at sizes 6–25 nm^2^, while treatment after 48 h BA cleared APP clusters, leading to a decrease in the number of APP clusters of all sizes (Figures 9C–E; Supplementary Figure S2).

### 3.8 Spermidine Reduces Lipid Peroxidation, and APP Protein Levels in the Hippocampus and Cortex in a Mouse Model of Paraquat-Induced Neuronal Injury

**FIGURE 10:**
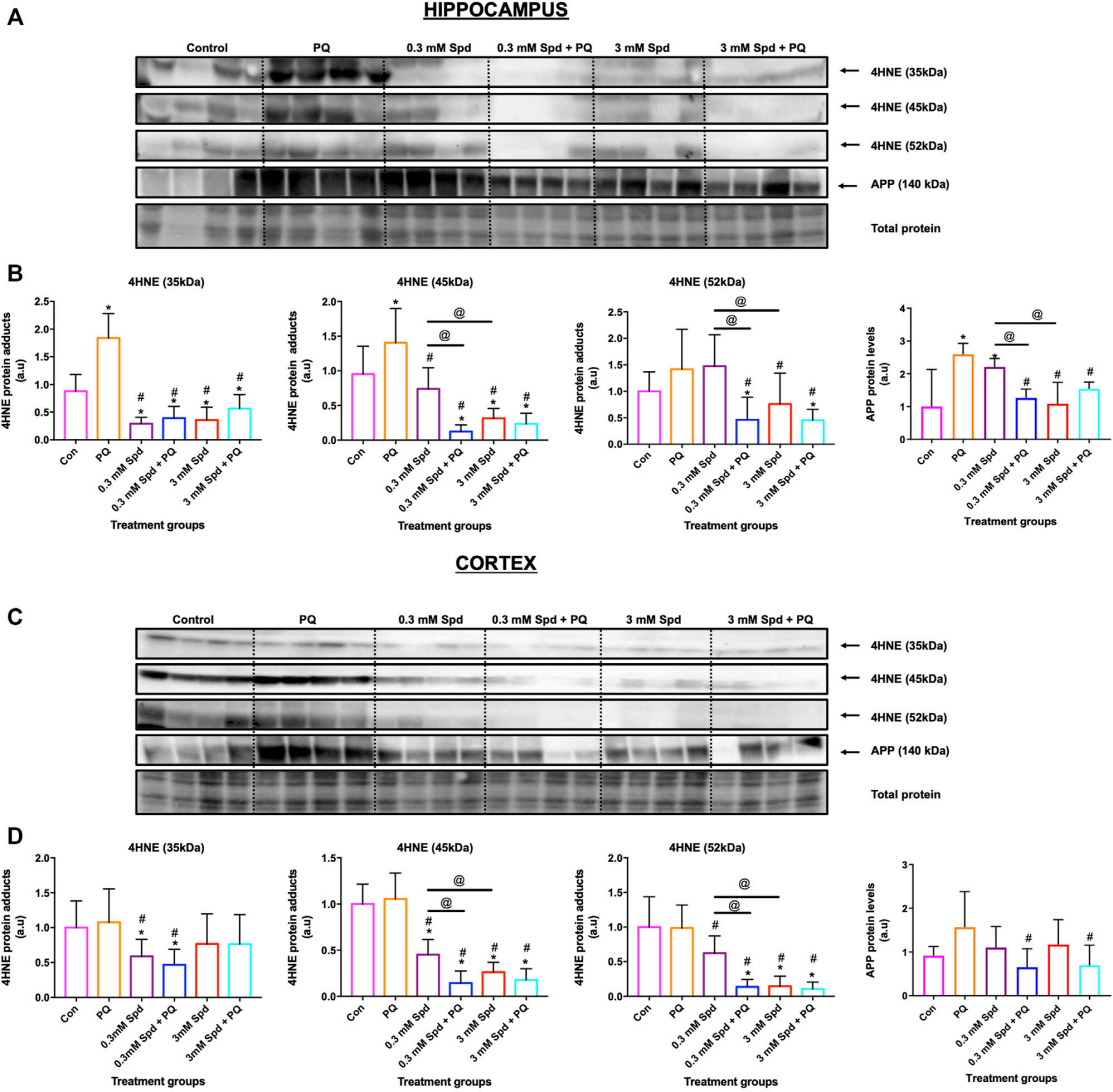
Spermidine reduces lipid peroxidation and amyloidogenic processing in the hippocampus and cortex region. Representative western blot and densitometric analysis of 4HNE adducts (35, 45 and 52 kDa) and APP protein levels in the hippocampus **(A and B)** and **(C and D)** cortex. The data are expressed as mean ± SEM. A total of 6–10 animals were used per group (*n* = 6–10). Spermidine in combination with PQ reduced 4HNE and APP protein levels compared to PQ treated group. * = *p* < 0.05 vs. control, # = *p* < 0.05 vs. PQ and @ = *p* < 0.05 vs. 0.3 mM Spd, arbitrary units (a.u).

To confirm the protective effects of spermidine against neuronal toxicity associated with neurodegeneration, a mouse model of paraquat-induced neuronal injury was used. We focussed on the hippocampus and cerebral cortex, the two regions impacted in the early and late stages of AD, respectively. Paraquat exposure resulted in a significant increase in lipid peroxidation end-product 4-hydroxy-2-nonenal (4HNE), a marker for oxidative stress, in the hippocampus (Figures 10A,B), but not in the cortex (Figures 10C,D). Similarly, a significant increase in APP protein levels was observed in the hippocampus, while no significant changes were detected in the cortex, suggesting injury susceptibility. Combination treatment of spermidine with PQ caused a significant reduction in 4HNE and APP protein levels in both regions, suggesting protective effects of spermidine in this context.

### 3.9 Differential Impact of Spermidine on Tubulin Acetylation and Autophagic Activity in the Hippocampus and Cortex in a Mouse Model of Paraquat-Induced Neuronal Injury

**FIGURE 11:**
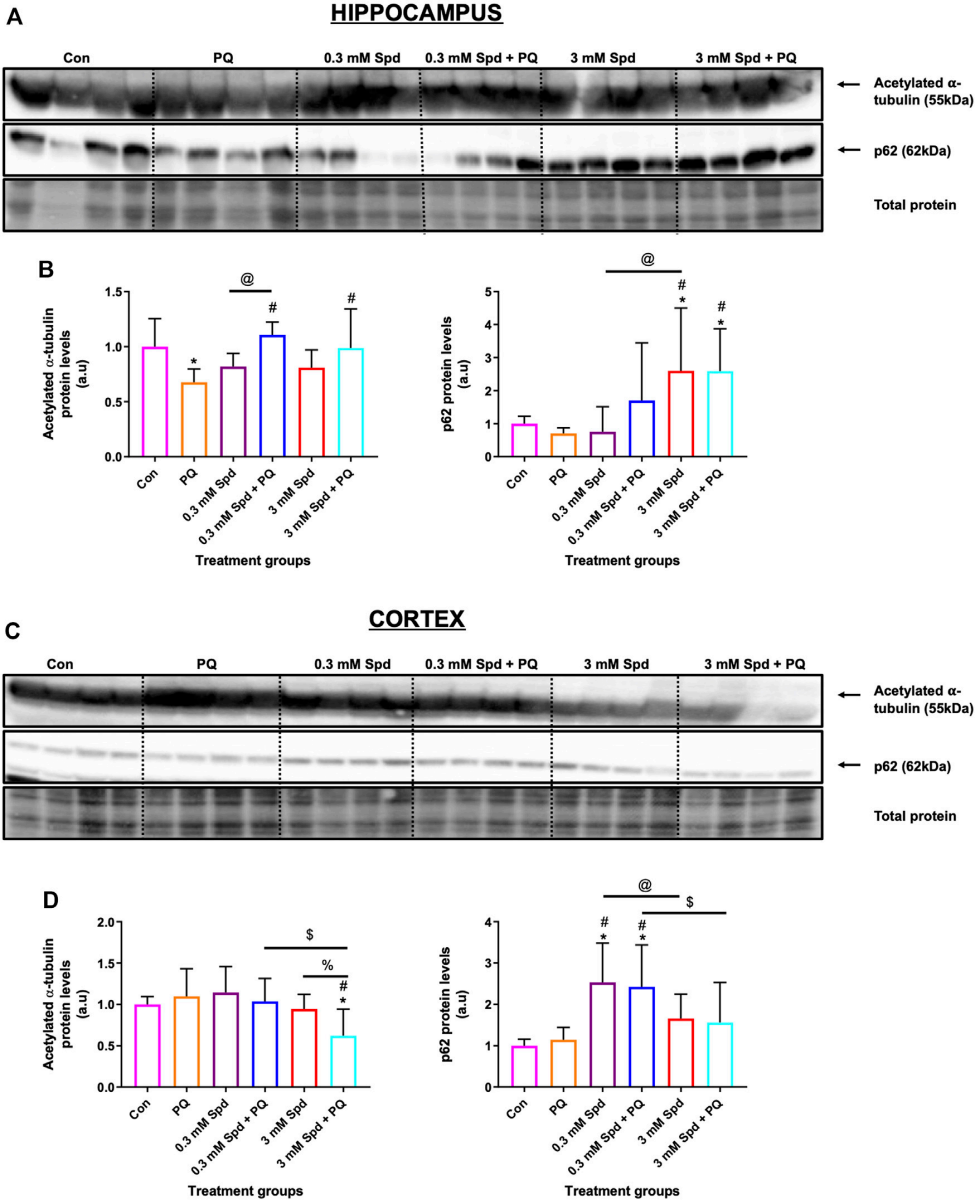
Differential effect of spermidine on tubulin acetylation and autophagic activity in the hippocampus and cortex. **(A,B)** Representative western blot and densitometric analysis of acetylated α-tubulin and p62 protein levels in the hippocampus and **(C,D)** cortex. The data are expressed as mean ± SEM. A total of 6–10 animals were used per group (*n* = 6–10). Spermidine in combination with PQ increased acetylated α-tubulin and p62 compared to PQ treated group in the hippocampus. In the cortex region, combination treatment of the low concentration spermidine increased p62 protein levels, while co-treatment with the high concentration of spermidine decreased acetylated α-tubulin with no effect on p62 levels. * = *p* < 0.05 vs. control, # = *p* < 0.05 vs. PQ, @ = *p* < 0.05 vs. 0.3 mM Spd, $ = *p* < 0.05 vs. 0.3 mM Spd + PQ and % = *p* < 0.05 vs. 3 mM Spd, arbitrary units (a.u).

Given the role of spermidine in autophagy and protein acetylation, we assessed the effects of spermidine on tubulin acetylation and p62 expression levels. Paraquat exposure resulted in a significant decrease in acetylated tubulin protein levels in the hippocampus (Figures 11A,B), while no significant changes were observed in the cortex (Figures 11C,D). Combination treatments of spermidine with paraquat in the hippocampus significantly increased acetylated α-tubulin protein levels compared to PQ treatment alone, while increasing the levels of p62 in a concentration-dependent manner. In the cortex region, combination treatment of the low concentration spermidine significantly increased p62 protein levels compared to PQ treatment alone, whereas co-treatment with the high concentration of spermidine significantly decreased acetylated α-tubulin while having no significant effect on p62 (Figures 11C,D).

## 4 Discussion

In this study, we revealed three critical aspects with regards to autophagy and cell death control. Firstly, a concentration-dependent effect of autophagy-inducing drugs on autophagic flux exists and its detection is highly dependent on the sensitivity of the method employed. Secondly, higher autophagic flux does not necessarily confer a higher degree of neuronal protection; and thirdly, susceptibility to PQ exposure and associated protection in the brain shows region specificity.

### 4.1 A Concentration-Dependent Autophagic Flux Exists Upon Spermidine Exposure

Although autophagy modulation using pharmacological agents has received considerable attention over the past years and although many autophagy modulating drugs are now known, it remains unclear to what extent different concentrations would change autophagic activity or autophagic flux, especially given the dynamic and cell-specific nature of protein degradation and proteolysis. One of the challenges in this context is to accurately assess and dissect the pathway intermediates i.e., autophagosomal and autolysosomal pool sizes and their turnover in a highly sensitive manner, to better understand the underlying mechanisms that govern a particular autophagic activity. Here, we report that a concentration-dependent effect on autophagy flux exists and that the detected change in flux is highly dependent on the sensitivity of the method used. Using western blotting, we found that only the high concentration of spermidine (10 μM) robustly induces autophagy activity (Figures 1A–C), while fluorescence-based analysis revealed that both low and high concentrations of spermidine (1 and 10 μM) enhance autophagy activity (Figure 2), whereas TEM analysis (i.e., number of AVs) showed that both the low and high concentration of spermidine do not enhance autophagic activity above basal levels (Figures 3A,C). These findings suggest that pool size assessment with FM is likely more sensitive. Indeed, FM allows for a relatively clear distinction between autophagosomes and autolysosomes using fluorescent labels which is not possible with western blotting or TEM, hence these structures in TEM are collectively called AVs. In addition, FM allows for imaging the whole cell using z-stacks, therefore providing data from the entire cell rather than one z-section from conventional TEM which was used here for EM-based quantification. This may indeed be a contributing factor why we observed no significant changes with TEM analysis (Figures 3A,C). Consistent with the FM results, our CLEM analysis supports the notion that 1 μM spermidine also induces autophagy activity as indicated by the increase in RFP-LC3ΔG positive structures in the 1 and 10 μM spermidine treated cells (Figure 4; Supplementary Figure S1) and further suggests that a heightened flux has led subsequently to engulfment of cytoplasmic cargo. Such observations have not yet been reported and enrich the autophagy field, indicating the temporal effects of autophagy activity on the clearance of cytoplasmic content. Although commonly used techniques such as western blotting and TEM provide invaluable information about autophagic activity i.e., autophagy protein, autophagosome abundance and cargo type contained in autophagosome, their use in isolation could result in the misinterpretation of data as they are not able to distinguish between autophagosomes and autolysosome pool size.

Moreover, we report that the size in vacuolar structures in response to the autophagy induction plays a major role in autophagic flux. EM allows observing different sizes in structures and autolysosomes, all of which allows to gain insights into cargo clearance and pool size dynamics. Although we did not see any differences in the sizes of AVs in the cells treated with only spermidine compared to the control cells, the combination of spermidine with BafA1 decreased the size of AVs compared to BafA1 alone, with 1 μM Spd + BafA1 reducing the size more compared to 10 μM Spd + BafA1 (Figures 3B,C). To our knowledge, this is the first study to report a reduction in the size of AVs upon spermidine treatment in the presence of BafA1 and is in contrast with our 3D-CLEM results (Figure 6). Morphometric analysis of reconstructed autophagosomes (Figure 6; Supplementary Figure S3) showed that spermidine treatment at a low concentration increases the volume and surface area of autophagosomes (Figure 6; Supplementary Video S2, Supplementary Figure S3), suggesting size regulation and effective cargo clearance. To our surprise, spermidine at a high concentration resulted in a decrease in the volume and surface area of autophagosomes, with a significant decrease seen in the surface area. These results suggest a concentration-dependent effect of spermidine on the volume and surface area and may highlight the importance of cargo-specific turnover in the context of a given autophagosome flux. Indeed, several studies have shown that Atg8 plays an important role in the size regulation of autophagosomes (Xie et al., 2008; Jin and Klionsky, 2014). In these studies, it was demonstrated that autophagy induction using rapamycin or nitrogen starvation elevates both ATG8 mRNA and its protein product, increasing the size of autophagosomes. Here, we suspect that spermidine might elicit similar mechanisms leading to an increase in autophagosome volume. Taken together, our data indicate how the mammalian system may respond to an autophagy modulator through both flux control as well as autophagosome size/volume control, thereby impacting cargo clearance. This deserves further study.

### 4.2 Higher Autophagic Flux Does Not Necessarily Lead to a Higher Degree of Protection

Neurons depend vastly on a high degree of efficient autophagic flux to maintain proteostasis. Modulation of autophagy using pharmacological agents is widely accepted as one of the major therapeutic interventions for age-related pathologies. Spermidine has been shown to induce autophagy in neurons, thus enabling effective clearance of aggregate prone proteins associated with neurodegeneration (Büttner et al., 2014; Sigrist et al., 2014; Bhukel et al., 2017; Yang et al., 2017). Despite the autophagy enhancing effects of spermidine, the impact of different concentrations in autophagy modulation and on subsequent protein clearance and neuronal toxicity is unclear. Here, we report that exposure to PQ leads to cellular toxicity, increased ROS production and loss of membrane integrity, while pre-treatment with spermidine at a low concentration (1 μM Spd) protected against the cytotoxic effects induced by PQ (Figure 7). PQ is a neurotoxicant associated with Alzheimer’s disease (Chen L. et al., 2012; Chen Y. W. et al., 2012; Jaroonwitchawan et al., 2017). It accumulates in the mitochondria, leading to ROS production (Jones and Vale, 2000; Yumino et al., 2002; Cochemé and Murphy, 2008) and cell death onset (Chun et al., 2001; González-Polo et al., 2004; Niso-Santano et al., 2006). Previous studies have shown that spermidine mediates protection against oxidative stress and cell death induced by H_2_O_2_ (Rider et al., 2007; Guo et al., 2011). Since spermidine is a known antioxidant, we speculate that it protects against ROS damage by increasing the generation of antioxidants enzymes such as superoxide dismutase (SOD) and glutathione peroxidase (GPX). In line with these findings, others have reported a decrease in PQ-induced ROS generation in SH-SY-5Y cells following pre-treatment with an antioxidant such as N-acetylcysteine (NAC) (Zhou et al., 2017) and curcumin (Jaroonwitchawan et al., 2017). Moreover, spermidine has been shown to serve dual roles, as it can also act as a substrate for enzymes responsible for ROS production (Murray Stewart et al., 2018). Thus, this might explain why pre-treatment with a high concentration of spermidine (10 μM Spd) failed to protect against ROS damage.

We also show that PQ exposure enhances the acetylation of α-tubulin compared to the control levels and that pre-treatment with low concentration of spermidine resulted in higher levels of acetylated α-tubulin compared to the PQ alone (Figure 8). These results suggest that PQ did not result in destabilization of microtubules but rather increased their stability, which appeared enhanced further in the presence of spermidine. We speculate that the increase in the stability of microtubules observed following PQ exposure might be the result of a stress response, likely in part due to autophagy activation. In support of this notion, various studies have reported that low concentrations of PQ stimulate characteristics of autophagy in *in vitro* models, with neurons ultimately succumbing to cell death (Niso-Santano et al., 2006; González-Polo et al., 2007a; González-Polo et al., 2007b; González-Polo et al., 2009). Due to the increased ROS generation, we observed following PQ treatment, this may suggest that PQ acts as a stressor that induces autophagy, probably in the initial stages, prior to mitochondrial dysfunction. Indeed, it has been shown that PQ induces an early ER stress response which was concurrent with activation of autophagy (González-Polo et al., 2007a; González-Polo et al., 2007b; Niso-Santano et al., 2011). Acetylation of microtubules is directly involved in autophagy as microtubules facilitate the transport of autophagosomes to lysosomes for degradation (Xie et al., 2010; Phadwal et al., 2018). Thus, spermidine increases the acetylation of tubulin and may facilitate autophagy degradation. In line with these findings, Phadwal et al. (2018) reported that spermine, a precursor of spermidine, increased tubulin acetylation resulting in the degradation of aggregated proteins in an *in vitro* model of Prion disease.

Next, we report in N2aSwe cells that APP overexpression induces cytotoxicity in a time-dependent manner, while spermidine at both concentrations protects against cytotoxicity induced by APP overexpression (Figure 9A). To our knowledge, this study is the first to demonstrate the protective effects of spermidine in an APP overexpression model. Administration of autophagy modulators has been shown to protect against APP-induced cytotoxicity effects in N2aSwe cells (Lee et al., 2015). Overproduction of APP results in increased levels of Aβ and APP C-terminal fragments (CTFβ), both of which contribute to the pathology of AD (Walsh and Selkoe, 2007). Here, we show that APP overexpression for 24 and 48 h led to an increase in APP protein levels (Figure 9B) and APP clusters in a time-dependent manner (Figures 9C–E). Interestingly, we noted that autophagy activity remained functional at both time points, with LC3-II and LAMP2A levels increasing in a time-dependent manner. Although spermidine did not reduce the levels of APP after 48 h of overexpression, both concentrations enhanced the clearance of APP clusters, thus reducing their number, however, the low concentration of spermidine was more effective. To our knowledge, this study is the first to assess APP clusters and their clearance by spermidine using dSTORM in models of AD and supports the findings obtained through WST-1 analysis. We speculate that spermidine protects against APP-induced toxicity by inducing autophagy (indicated by the increase in LC3-I levels), thereby shifting the equilibrium of soluble and aggregated APP. Overall, this study contributes to the current body of literature, demonstrating that it must be considered which drug concentration to use, to better align flux control with the most favourable autophagy activity in the context of autophagy dysfunction.

### 4.3 Susceptibility to PQ in the Brain Is Region Specific

Although the mechanisms of action of PQ in AD are not fully elucidated, it is well known that PQ exerts its toxicity by inducing oxidative stress and mitochondrial damage (Lin and Beal, 2006; Drechsel and Patel, 2008; Baltazar et al., 2014), both of which have been implicated in the pathogenesis of AD (Lin and Beal, 2006; Chen L. et al., 2012). Multiple studies have reported high levels of lipid peroxidation, a marker for oxidative stress in the brains of patients with AD (Zhao and Zhao, 2013; Wang et al., 2014). Here, we report that PQ-induced toxicity impacts the brain regions differentially, with the hippocampus being highly susceptible to PQ-induced injury (Figure 10). Exposure to PQ enhanced lipid peroxidation and APP protein levels in the hippocampus, but not in the cortex, suggesting injury susceptibility. In addition, we show that PQ treatment resulted in the destabilization of microtubules i.e., a decrease in acetylated tubulin, in the hippocampus, but not in the cortex (Figure 11). These results may point towards region-specificity and are in line with the findings that the pathological changes of protein aggregation and neuronal loss are manifesting first in the hippocampus and later in the cortex, as the disease pathology progresses (Braak and Braak, 1991; Braak et al., 2004; Braak and Del Tredici, 2012). Both regions were favourably impacted by spermidine treatment in drinking water (0.3 mM Spd + PQ and 3 mM Spd + PQ) as evident by a decrease in 4HNE and APP protein levels, supporting the notion of a supplement-based adjuvant therapy. In addition, spermidine treatment rescued PQ-induced destabilization of the microtubules in the hippocampus but not in the cortex. Spermidine is known to increase the acetylation of tubulin by suppressing EP300, and in turn stimulate autophagic flux in this manner (Pietrocola et al., 2015; Madeo et al., 2018). Increased acetylation of microtubules facilitates the retrograde mobilization of autophagosomes from the periphery to the lysosomes localised in the cytoplasm for degradation (Xie et al., 2010; Phadwal et al., 2018). Therefore, we hypothesise that spermidine increases the acetylation of tubulin by suppressing EP300, leading to an increased autophagic flux, where toxic protein and damaged organelles induced by PQ treatment are engulfed in the autophagosomes and transported to the lysosomes for subsequent degradation. In support of this notion, here we report that spermidine at both dosages upregulated autophagy activity i.e., increase p62 levels in a concentration-dependent manner in the hippocampus, while only the low dose increased p62 in the cortex. Taken together, our findings shed light on the mechanisms of spermidine induced neuronal protection in this model system. Due to the importance of the hippocampus in neurodegeneration and memory formation, these results are of significance and point towards spermidine as a potential therapeutic agent for Alzheimer’s disease.

## 5 Conclusion

Taken together, it becomes clear that autophagy assessment requires the use of multiple tools as not all techniques are sufficiently sensitive to detect differences in flux, with effects manifesting differently under control and pathological conditions, depending on the injury model, cell type, and model system employed. We provide evidence of the distinct, context dependent protective role of spermidine in an *in vitro* model of APP overexpression as well as *in vitro* and *in vivo* models of PQ-induced neuronal toxicity, suggesting that the cell type, nature of the injury, brain region and the drug concentration utilised should be critically considered when performing drug screening so that a given concentration can be most favourably aligned to offset autophagy dysfunction. It is widely known that neurons depend vastly on a high degree of efficient autophagic flux to survive due to their post-mitotic nature, high protein synthesis rate and energy demands. However, protein degradation and proteolysis are regional and cell-specific, requiring sensitive characterization of autophagic flux, autophagosomal and lysosomal pool sizes and transition time to better understand the underlying mechanisms that govern a particular autophagic activity. While extensive data from preclinical studies provide strong evidence that autophagy upregulation provides therapeutic benefits for the treatment of AD, numerous unanswered questions remain regarding the efficiency of autophagy induction using such interventions. Therefore, a better understanding is required regarding the context of neuronal protection, including a quantitative approach to assess autophagy. This is critical in the context of brain-specific spatio-temporal autophagic activity in physiology and neuronal pathology, to better regulate autophagy and thereby control neuronal fate. In addition, autophagy modulation requires careful alignment with aggregate-prone protein cargo levels as well as markers of disease, to precision-target the autophagic machinery according to defect localization. Here, we have utilised dSTORM to assess APP clusters and 3D CLEM to assess autophagosome volume as a point of departure in this regard. Future work may, therefore, focus on improved methodologies, including high throughput-based, multi-scale approaches to advance translation. Our results suggest that the administration of spermidine may represent a favourable therapeutic strategy for the treatment of AD. Further studies using transgenic mouse models of APPSwe, PS1 and 3xTg AD are warranted to further verify the protective effects of spermidine. In such models, behavioural studies assessing cognitive function will provide invaluable information about the role of spermidine in learning and memory. Applications with this approach within the *in vitro* and *in vivo* environment will undoubtedly allow improved and progressive translation of autophagy control in preclinical and clinical settings and may advance autophagy modulation as a prime candidate for the treatment of neurodegeneration.

## Data Availability

The original contributions presented in the study are included in the article/Supplementary Material, further inquiries can be directed to the corresponding author.

## Supplementary Material

Supplementary Figure S1 Spermidine induces autophagy. SR-SIM micrographs showing the overview of GT1-7 cells expressing GFP-LC3-RFP-LC3ΔG after treatments. (Top panel) spermidine (1 and 10 μM) induces the accumulation of RFP-LC3ΔG positive structures compared to the control (untreated) cells, suggesting heightened autophagic flux. (Bottom panel), spermidine in combination with BafA1 induces the accumulation of GFP-LC3 positive structures compared to BafA1 treated group, suggesting an induced flux. Scale bar: 3 μm.

Supplementary Figure S2 Spermidine reduces the size and number of APP clusters upon APP overexpression. Representative dSTORM micrographs in Gauss mode showing the localisation of APP clusters in the whole cell and their clearance by spermidine. Scale bar: 1 μm.

Supplementary Figure S3 3D model of autophagosomes. Overview of reconstructed autophagosomes from FIB-SEM stack showing different sizes of autophagosomes in the control (untreated) and BafA1 group (Left), 1 μM Spd and 1 μM Spd + BafA1 (middle) and 10 μM Spd & 10 μM Spd + BafA1 (right). Scale bar: 50 nm.

Supplementary Video S1 3D Correlative SR-SIM and FIB-SEM of 1 μM Spd treated cell showing the localisation of GFP-LC3 and RFP-LC3ΔG positive structures in vacuolar structures of high electron density. Both GFP-LC3 and RFP-LC3ΔG decorate the conglomerate structure. RFP-LC3ΔG localises inside the conglomerate structure as part of the cargo.

Supplementary Video S2 3D Correlative SR-SIM and FIB-SEM of 1 μM Spd treated cell. GT 1-7 neuronal cell expressing GFP-LC3-RFP-LC3ΔG is shown in relation to its rendered autophagosomes using FIB-SEM data. Segmentation of autophagosomes highlight large volume vesicles.
