# Supplementary figures and images for: Investigating the Role of Spermidine in a Model System of Alzheimer’s Disease Using Correlative Microscopy and Super-resolution Techniques
Source: Front Cell Dev Biol. 2022 May 17;10:819571. doi: 10.3389/fcell.2022.819571 (PMC9152225; doi:10.3389/fcell.2022.819571)

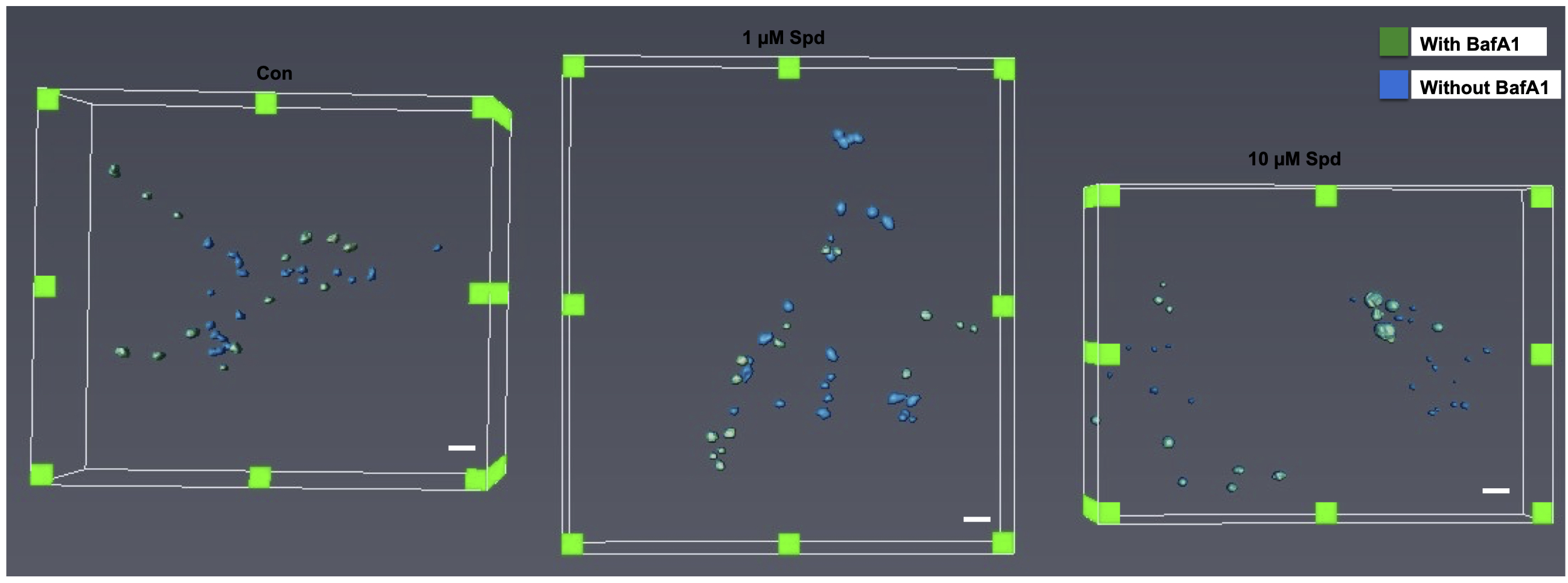

Supplement: Supplementary file 1 [file Image3.JPEG]

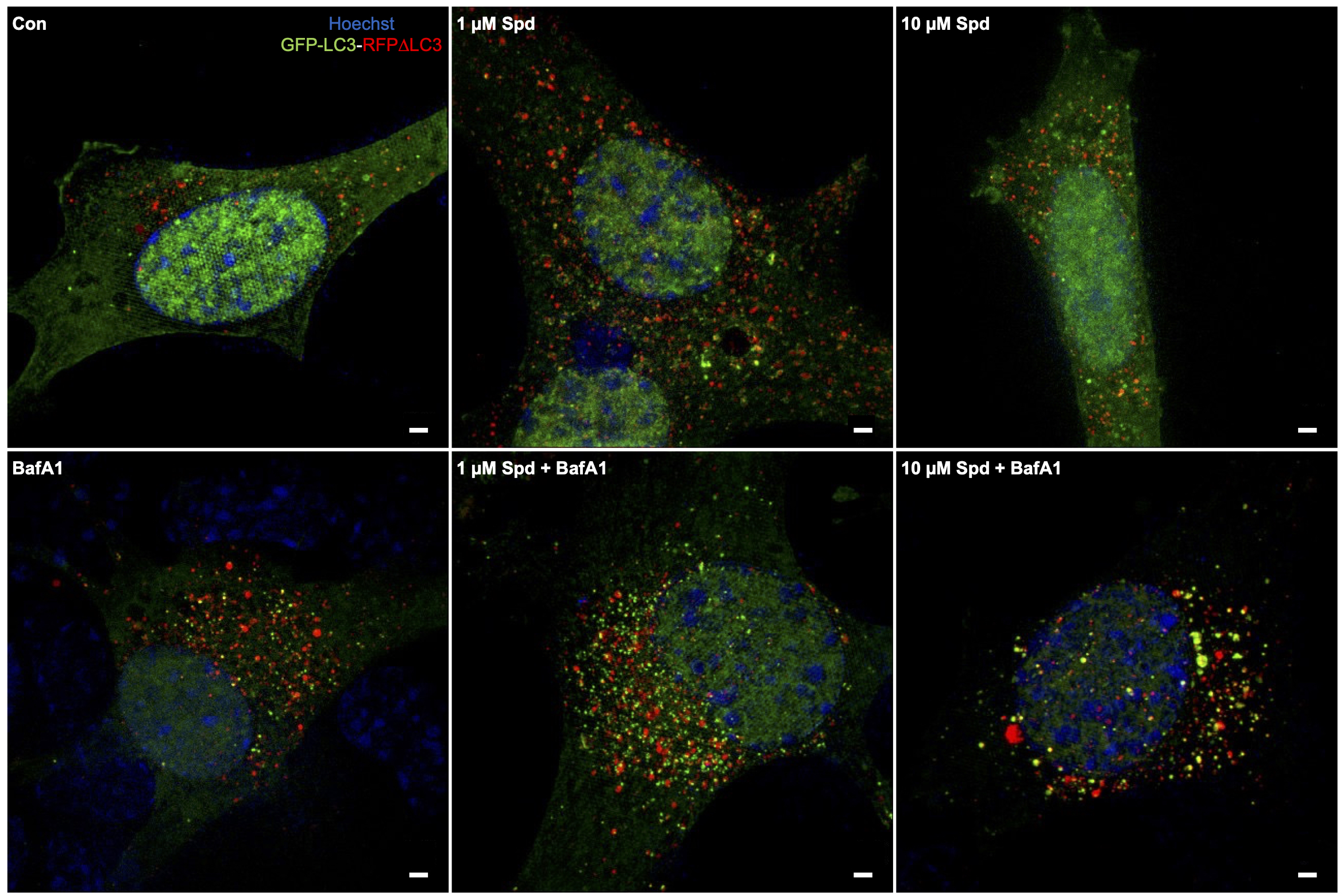

Supplement: Supplementary file 2 [file Image1.JPEG]

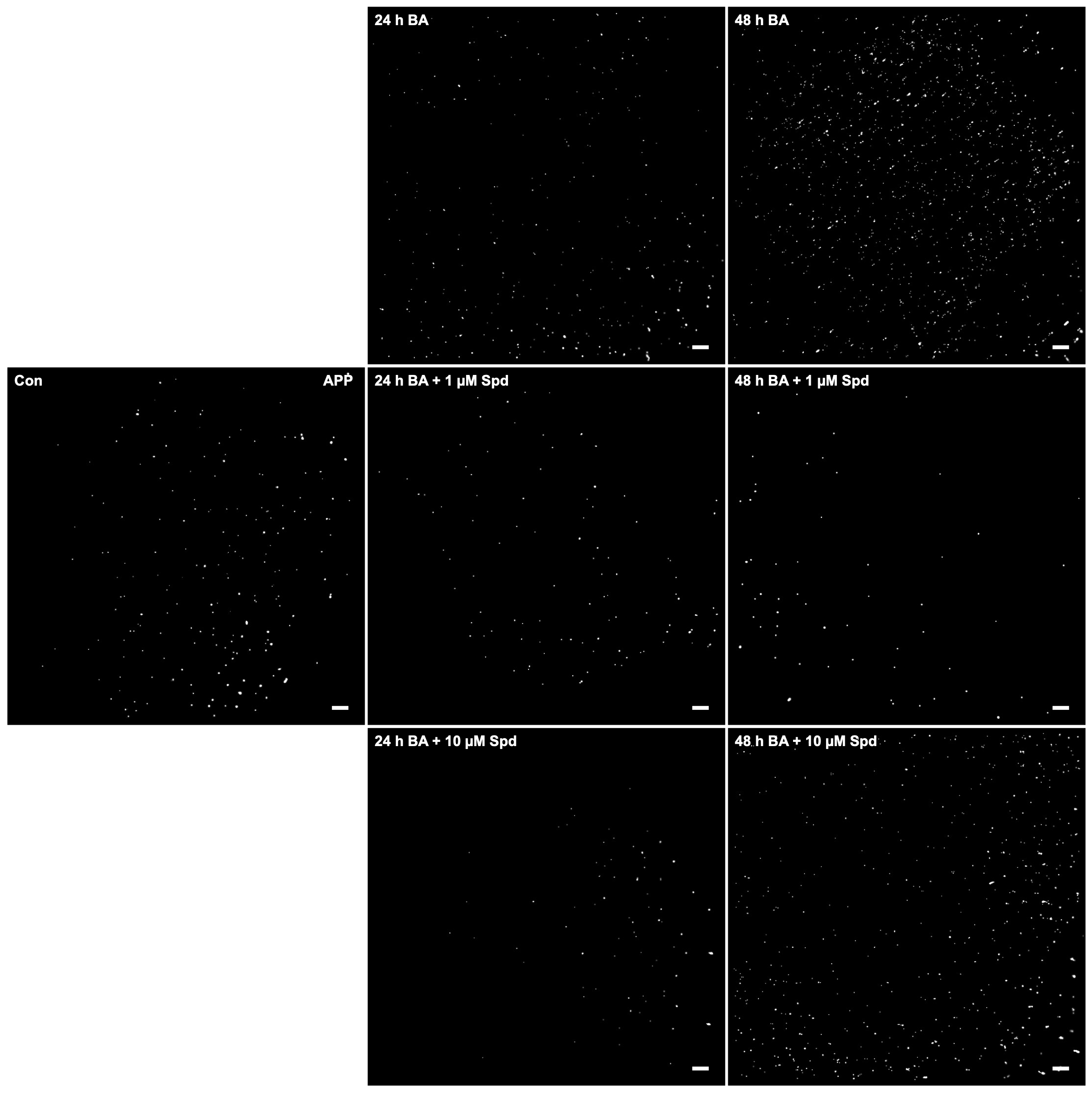

Supplement: Supplementary file 4 [file Image2.JPEG]
